# Deficiency of the zinc finger protein ZFP106 causes motor and sensory neurodegeneration

**DOI:** 10.1093/hmg/ddv471

**Published:** 2015-11-24

**Authors:** Peter I. Joyce, Pietro Fratta, Allison S. Landman, Philip Mcgoldrick, Henning Wackerhage, Michael Groves, Bharani Shiva Busam, Jorge Galino, Silvia Corrochano, Olga A. Beskina, Christopher Esapa, Edward Ryder, Sarah Carter, Michelle Stewart, Gemma Codner, Helen Hilton, Lydia Teboul, Jennifer Tucker, Arimantas Lionikas, Jeanne Estabel, Ramiro Ramirez-Solis, Jacqueline K. White, Sebastian Brandner, Vincent Plagnol, David L. H. Bennet, Andrey Y. Abramov, Linda Greensmith, Elizabeth M. C. Fisher, Abraham Acevedo-Arozena

**Affiliations:** 1 MRC MammalianGenetics Unit, Harwell, OxfordshireOX11 0RD, UK,; 2 UCL Institute ofNeurology and MRC Centre for Neuromuscular Disease, Queen Square, LondonWC1N 3BG, UK,; 3 Health Sciences, University of Aberdeen, AberdeenAB25 2ZD, UK,; 4 Nuffield Department of Clinical Neurosciences, University of Oxford, OxfordOX3 9DU, UK,; 5 Sanger Institute, Wellcome Trust Genome Campus, Hinxton, CambridgeshireCB10 1SA, UK and; 6 UCL Genetics Institute, LondonWC1E 6BT, UK

## Abstract

Zinc finger motifs are distributed amongst many eukaryotic protein families, directing nucleic acid–protein and protein–protein interactions. Zinc finger protein 106 (ZFP106) has previously been associated with roles in immune response, muscle differentiation, testes development and DNA damage, although little is known about its specific function. To further investigate the function of ZFP106, we performed an in-depth characterization of *Zfp106* deficient mice (*Zfp106^−/−^*), and we report a novel role for ZFP106 in motor and sensory neuronal maintenance and survival. *Zfp106^−/−^* mice develop severe motor abnormalities, major deficits in muscle strength and histopathological changes in muscle. Intriguingly, despite being highly expressed throughout the central nervous system, *Zfp106^−/−^* mice undergo selective motor and sensory neuronal and axonal degeneration specific to the spinal cord and peripheral nervous system. Neurodegeneration does not occur during development of *Zfp106^−/−^* mice, suggesting that ZFP106 is likely required for the maintenance of mature peripheral motor and sensory neurons. Analysis of embryonic *Zfp106^−/−^* motor neurons revealed deficits in mitochondrial function, with an inhibition of Complex I within the mitochondrial electron transport chain. Our results highlight a vital role for ZFP106 in sensory and motor neuron maintenance and reveal a novel player in mitochondrial dysfunction and neurodegeneration.

## Introduction

ZFP106 is a zinc finger protein with proposed roles in transcriptional control, RNA metabolism, the immune response, muscle development and differentiation and testes development (1–3). Phosphorylation of ZFP106 increases following DNA damage, which might suggest a possible role in the DNA damage response pathway (4). Recently, ZFP106 has been identified as a novel factor regulating transcription initiation by targeting RNA-polymerase I to the promoter of ribosomal RNA genes (5), linking for the first time ZFP106 function and RNA metabolism. Interestingly, the human gene encoding ZFP106 (*ZFN106*) is located within a familial amyotrophic lateral sclerosis (ALS) locus (ALS5) on human chromosome 15, while human ZFP106 is proposed to localize to the nucleolus (1).

Z*fp106* encodes a 1888-amino acid protein with two N-terminal C_2_H_2_ zinc finger motifs, two C-terminal CWCH_2_ zinc finger motifs and seven WD40 repeats (2,3,6). Zinc finger motifs are important for protein–protein interactions and nucleic-acid binding (7), whilst WD40 repeats are involved in protein–protein interaction and facilitate the formation of large multi-protein complexes (8). Putative orthologues of *Zfp106* are only found in mammals and exhibit a high degree of conservation between species.

*Zfp106* mRNA expression is proposed to be driven from two promoters, with the resulting transcripts producing a number of isoforms (2). Full-length *ZFP106* (*ZNF106*) mRNA is expressed ubiquitously and expression is particularly high in the heart, skeletal muscle and testis (2). Nuclear respiratory factor-1 (NRF-1) can activate *Zfp106* mRNA expression, with expression patterns of full-length *Zfp106* and *Nrf1* mRNA coinciding in mouse embryos (2). A short isoform is specifically expressed in skeletal muscle, regulated by myogenin, and is strongly upregulated during myogenic differentiation (2).

While previous research on ZFP106 indicates different functions, in this study we have created the first ZFP106 animal model to further understand its *in vivo* functions. We have taken advantage of the resource provided by the international Knock Out Mouse Project (KOMP) (9) and the Sanger Mouse Genetics Project to further elucidate *Zfp106* function. Mice homozygous for the knockout first promoter-less allele (tm1a(KOMP)Wtsi) in *Zfp106* develop severe gait and motor abnormalities that deteriorate with age. The motor abnormalities exhibited by *Zfp106^−/−^* mice are likely due to a severe progressive adult-onset degenerative sensory-motor axonopathy.

## Results

### *Zfp106* deficiency causes progressive motor abnormalities

**Figure 1.**
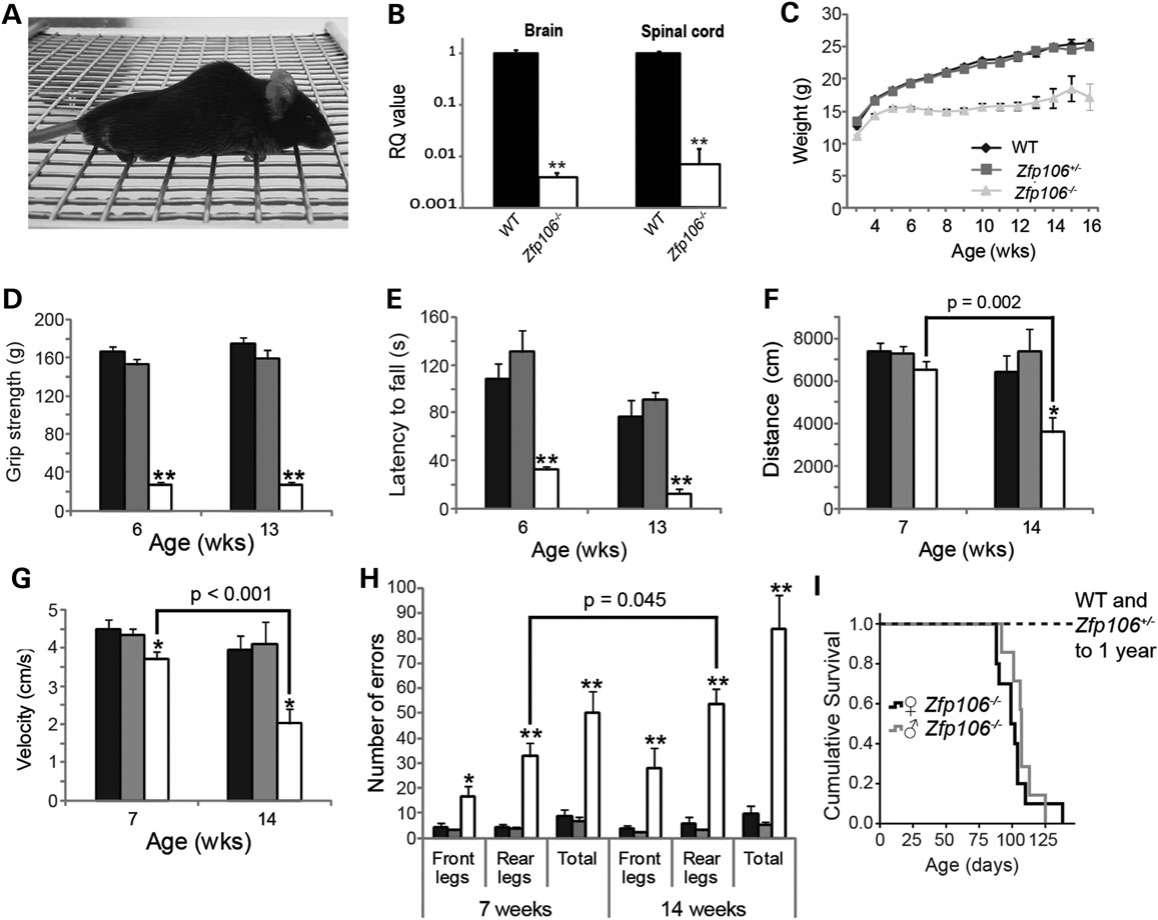
Behavioural analyses of *Zfp106^−/−^* mice reveal severe motor abnormalities. Black bars: *Zfp106^−/−^*, grey bars: *Zfp106^+/−^*, white bars: *Zfp106^−/−^*. (**A**) Photograph of mutant *Zfp106^−/−^* mouse at 15-weeks of age displaying pronounced kyphosis and unable to coordinate hind limbs on wire grate. (**B**) qPCR analysis of *Zfp106* expression levels in brain and spinal cord of 6-week-old male WT and *Zfp106^−/−^* littermates (*n* = 3 per genotype); expression in *Zfp106^−/−^* mice is normalized to *Zfp106* expression in respective WT tissues, taken as a value of 1 (Log scale). (**C**) Female weights recorded weekly from ages 3 to 15 weeks; at least five mice were assessed per genotype per time point. Weight is diminished in *Zfp106^−/−^* female mice from 3-weeks of age (*P* = 0.04) and continues to significantly decrease, compared with WT and *Zfp106^+/−^* animals, to 15-weeks of age (*P* < 0.001). (**D**) Female grip strength and (**E**) accelerated Rotarod performance are reduced in *Zfp106^−/−^* mice at 6- and 13-weeks of age compared with WT and *Zfp106^+/−^* littermates (*n*≥ 6 per genotype). (**F** and **G**) Open field assessment of (F) distance moved, and (G) velocity for female mice at 7- and 14-weeks of age (see ‘Materials and Methods’). A reduction in distance moved (F), and velocity (G), was seen in 14-week-old female *Zfp106^−/−^* mice when compared with 7-week female *Zfp106^−/−^* mice; *P* values are indicated (*n* ≥ 5 per genotype and time point) and 14-week old WT littermates. (**H**) Female mice assessed for defects in coordination of front and rear legs at 7- and 14-weeks of age using the Locotronic^®^ system (see ‘Materials and Methods’). *Zfp106^−/−^* mice made significantly more front and rear leg placement errors compared with WT littermates. Rear leg errors also significantly increased for 14-week-old *Zfp106^−/−^* mice compared with 7-week-old *Zfp106^−/−^* mice (*n* ≥ 5 per time point and genotype; *P* value indicated). Numbers and percentages shown represent the mean ± SEM. *P < 0.05; **P < 0.001. (**I**) Survival of male and female *Zfp106^−/−^* mice as determined by their humane endpoint (see ‘Materials and Methods’).

A knockout first promoter-less allele (tm1a(KOMP)Wtsi) within *Zfp106* was created by The Sanger Mouse Genetics Project (http://www.sanger.ac.uk/mouseportal/) through KOMP (9). The construct targets *Zfp106* intron 1 on chromosome 2 and includes a β-galactosidase (*LacZ*) reporter allele (see ‘Materials and Methods’). Mice homozygous for the tm1a(KOMP)Wtsi allele in *Zfp106* (*Zfp106^−/−^*) are born in Mendelian ratios (data not shown) and both *Zfp106*^−/−^male and female mice develop an abnormal gait from ∼3 weeks of age, which deteriorates with age, causing animals to inaccurately place their limbs, especially their hindlimbs, when walking across a wire grate (Supplementary Material, Movie S1). *Zfp106^−/−^* animals also become severely kyphotic and develop limb grasping defects at 15-weeks of age whereby they pull all of their limbs into their body (Fig. 1A and Supplementary Material, Movie S1).

To assess *Zfp106* expression we performed whole-genome transcriptomic analysis (RNA-seq) on spinal cord and qPCR on spinal cord and brain from adult wild-type (WT) mice and *Zfp106^−/−^* littermates. The unbiased RNA-seq expression data show that whilst some *Zfp106* expression remains in spinal cords of *Zfp106^−/−^* mice, the expression levels are <20% of those identified in WT littermates (Table 1). qPCR of *Zfp106* also revealed reduced expression in *Zfp106^−/−^* mouse tissues when compared with WT littermates; however, analysis via this technique indicated lower *Zfp106* expression in *Zfp106^−/−^* mice when compared with RNA-seq analysis (0.4 and 0.7% of WT levels for brain and spinal cord, respectively) (Fig. 1B). This reduction in *Zfp106* expression was also confirmed via northern-blot analysis from both brain and spinal cord (Supplementary Material, Fig. S1). The muscle-specific transcript was also downregulated in *Zfp106*^−/−^ muscle via qPCR (Supplementary Material, Fig. S1). We also confirmed the expression pattern of *Zfp106* using the *LacZ* reporter contained within the *Zfp106* knockout cassette, with expression during embryogenesis and in many tissues in adulthood, including the central nervous system (CNS) and hind limb muscle (Supplementary Material, Fig. S1).

**Table 1.** Top 10 statistically significant (adjusted *P* value <1E − 14) positively and negatively regulated genes from *Zfp106*^−/−^ spinal cords via RNA-seq

Gene	Fold difference
*Calcoco2*	0.15
*Zfp106*	0.19
*Chodl*	0.38
*Calca*	0.55
*Pla2g3*	0.60
*Slc10a4*	0.61
*Mmp9*	0.62
*Aif1l*	0.67
*Dhcr24*	0.69
*Nefh*	0.76
*Mmp12*	44.58
*Cst7*	19.97
*Atp6v0d2*	18.68
*Cxcl10*	13.25
*Cxcl9*	12.88
*Itgax*	12.23
*Tgm1*	11.89
*Gpnmb*	11.63
*Lgals3*	10.13
*Igfbpl1*	13.25

We evaluated the behavioural phenotypes of male (Supplementary Material, Fig. S2) and female *Zfp106^−/−^* cohorts (Fig. 1) and found no sex differences. Body weight of female *Zfp106^−/−^* mice are significantly reduced from 3 weeks of age compared with WT and *Zfp106^+/−^* littermates (Fig. 1C; *P* = 0.04). When comparing grip strength of WT, *Zfp106^+/−^* and *Zfp106^−/−^* females we found a dramatic reduction in *Zfp106^−/−^* mice at 6 and 13 weeks of age (Fig. 1D). Rotarod testing revealed a similarly reduced motor performance in *Zfp106^−/−^* females compared with WT littermates (Fig. 1E). We also examined the behaviour of 6-week-old WT, *Zfp106^+/−^* and *Zfp106^−/−^* littermates using a modified SHIRPA analysis, which assesses a battery of simple phenotypic traits, with an emphasis on neurological function (10,11). These tests included: the presence/absence of tremors, limb grasping of front or rear legs, gait, the ability to hang from a wire (wire manoeuvre), and the ability to walk down a vertical wire grate (negative geotaxis, see ‘Materials and Methods’). Table 2 summarizes SHIRPA analysis from 6-week-old WT, *Zfp106^−/+^* and *Zfp106^−/−^* littermates. ∼66% *Zfp106^−/−^* mice display mild to moderate tremors, >50% display limb grasping defects and all *Zfp106^−/−^* mice are severely impaired in wire hanging and are unable to maintain grip when made to walk down a vertical wire grate (negative geotaxis).

**Table 2.** Movement and neurological associated defects in *Zfp106^−/−^* mice, assessed using modified SHIRPA analysis

Genotype	Sample size^a^	Tremors (%)^b^	Limb grasp (%)^b^	Wire Manoeuvre deficits (%)^b^	Negative geotaxis deficits (%)^b^
WT	6	0	0	0	0
*Zfp106^+/−^*	7	0	0	0	0
*Zfp106^−/−^*	9	66.7	55.6	100	100

^a^Female animals assessed at 6 weeks of age.

^b^% of animals that displayed defects in each of the semi-quantitative measures, assessed during the modified SHIRPA test (see ‘Materials and Methods’).

WT, *Zfp106^+/−^ and Zfp106^−/−^* littermates were assessed in an open field paradigm, whereby mouse movement is tracked within an arena for 30 min. We observed a progressive deterioration in the distance moved (Fig. 1F), the mean velocity of movement (Fig. 1G), and the percentage of time spent moving (data not shown) between 7- and 14-week-old *Zfp106^−/−^* mice. To characterize these progressive motor abnormalities further we analysed the number of placement errors made by either their front or rear legs using the Locotronic^®^ system (Fig. 1H). At 7 weeks of age *Zfp106^−/−^* mice produce significantly more front and rear leg errors compared with WT littermates (front leg errors, WT 4.4 ± 1.4 versus *Zfp106^−/−^* 16.6 ± 4.3, *P* < 0.05; rear leg errors, WT 4.2 ± 1.1 versus *Zfp106^−/−^* 32.8 ± 5.2; *P* < 0.001). Importantly, the number of rear leg errors significantly increases in *Zfp106^−/−^* mice at 14 weeks of age to 53.5 ± 6.4 (*P* = 0.045) compared with 7-week-old *Zfp106^−/−^*, showing an age-dependent deterioration in motor coordination.

*Zfp106^+/−^* mice did not display any differences from WT littermates in any of the behavioural tests outlined above. Five one-year old *Zfp106^+/−^* mice were also analysed by modified SHIRPA with no obvious defects detected (data not shown).

### *Zfp106^−/−^* mice have a reduced lifespan

Lifespan of *Zfp106^−/−^* mice was determined as the time to reach humane endpoint, defined as the time taken to lose 20% of peak body weight or an inability to right themselves within 20 s when placed on their sides. Out of 17 *Zfp*^−/−^ mice examined, the majority were culled due to a 20% loss of peak body weight (*n* = 12), whilst a smaller proportion were culled due to an inability to right themselves impinging on their ability to feed (*n* = 5). All *Zfp106^−/−^* mice exhibited severe locomotor deficits at humane endpoint. The average lifespan of female *Zfp106^−/−^* mice was 102 ± 5 days (*n* = 10) and for males was 107 ± 4 days (*n* = 7) (Fig. 1I); there was no significant difference in survival between *Zfp106^−/−^* males and females (data not shown). WT and *Zfp106^+/−^* littermates survived with no abnormalities up to 365 days.

### Hind limb muscle physiology of early and late stage *Zfp106^−/−^* mice

**Figure 2.**
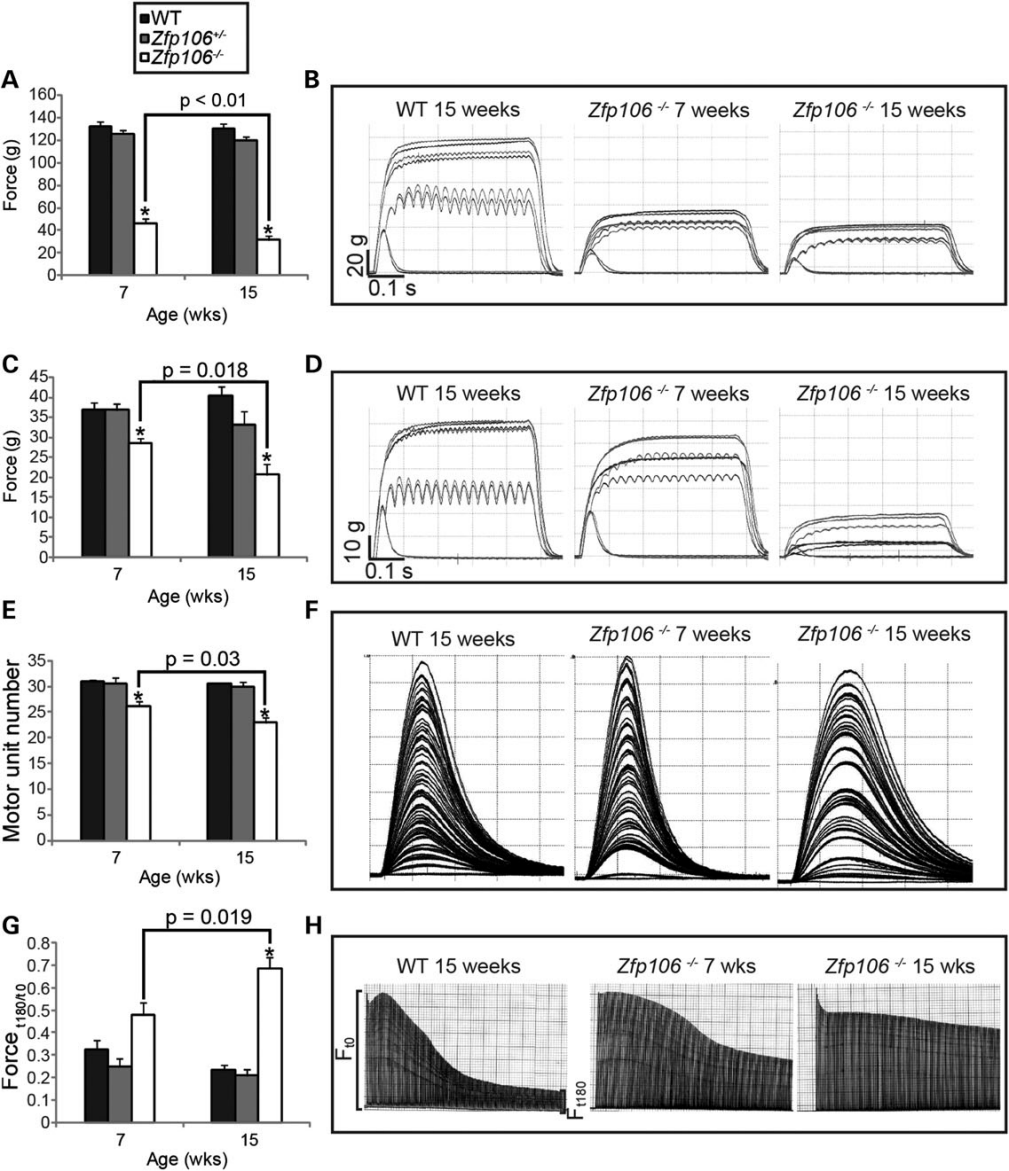
Assessment of hind limb muscle force, motor unit survival and muscle fatigue on female mice. (**A** and **B**) Summary of the maximum tetanic muscle force generated from the TA muscle. (A) TA tetanic muscle force generated by *Zfp106^−/−^* mice at 7-weeks of age (47 g ± 4 g) is reduced compared with WT littermates (133 g ± 4 g). TA tetanic tension is further reduced in 15-week-old *Zfp106^−/−^* mice (32 g ± 4 g) compared with 7-week-old *Zfp106^−/−^* mice. (B) Representative traces of TA tetanic tension from WT and *Zfp106^−/−^* mice at 7- and 15-weeks of age. (**C** and **D**) Tetanic muscle force generated from the EDL at 7- and 15-weeks of age (see ‘Materials and Methods’). (C) EDL tetanic muscle force generated by *Zfp106^−/−^* mice at 7-weeks (29 g ± 1 g) is reduced compared with WT littermates (37 g ± 2 g) and becomes further reduced in 15-week-old *Zfp106^−/−^* mice (21 g ± 3 g). (D) Representative traces of EDL tetanic tension from WT, 7- and 15-week old *Zfp106^−/−^* mice. (**E** and **F**) The number of surviving motor units in the EDL muscle of female mice at 7- and 15-weeks of age; motor units are reduced in 7-week-old *Zfp106^−/−^* mice (26 ± 0.9) compared with WT littermates (31 ± 0.4), and are further reduced in 15-week-old *Zfp106^−/−^* mice (23 ± 1). (F) Representative motor unit traces from EDL muscles of WT, 7- and 15-week old *Zfp106^−/−^* mice. Each twitch trace recording is a single motor unit. (**G** and **H**) FI (see ‘Materials and Methods’ section) of EDL muscle for female mice at 7 and 15-weeks of age. FI of 7-week old *Zfp106^−/−^* mice (0.48 ± 0.05) is increased compared with WT littermates (0.32 ± 0.04) but not significantly (*P* = 0.07). FI increases further in 15-week-old *Zfp106^−/−^* mice (0.69 ± 0.05) compared with 7-week old *Zfp106^−/−^* mice. (H) Representative fatigue traces from WT, 7- and 15-week *Zfp106^−/−^* mice, produced by repetitive stimulation of the EDL muscle for 180 s. Each line in the trace represents a single tetanic tension. Results are presented as the mean ± SEM. At least 10 legs were assessed per genotype per time point. *P ≤ 0.001, or indicated within each chart when comparing *Zfp106^−/−^* mice from 7- and 15-weeks of age.

To further characterize the motor abnormalities in *Zfp106^−/−^* mice, we analysed *in vivo* the physiological characteristics of the fast twitch hind limb muscles tibialis anterior (TA) and extensor digitorum longus (EDL). The maximum isometric force (tetanic tension) was measured in the TA and EDL muscles from both legs of 7- and 15-week-old WT, *Zfp106^+/−^* and *Zfp106^−/−^* littermates (Fig. 2A–D). The maximum force generated from the TA of 7-week-old *Zfp106^−/−^* mice was ∼65% less than WT littermates and further reduced to ∼75% of WT littermates at 15-weeks of age (Fig. 2A and B). Similarly, the tetanic tension of EDL muscles from 7-week-old *Zfp106^−/−^* mice was ∼20% less than WT littermates and reduced by ∼50% in 15-week-old *Zfp106^−/−^* mice compared with WT littermates (Fig. 2C and D). Thus, TA muscles are affected earlier and more severely than the EDL muscles, as has been previously reported in mouse models of other neuromuscular disorders (12). Interestingly, both TA and EDL muscles displayed a clear deterioration in the amount of force generated as *Zfp106^−/−^* mice aged.

We next determined the number of functional motor units innervating EDL muscles in 7- and 15-week-old WT, *Zfp106^+/−^* and *Zfp106^−/−^* mice (Fig. 2E and F). In 7-week-old *Zfp106^−/−^* mice, there was a small but significant reduction in motor unit survival, such that 26 ± 0.9 motor units (*n* = 12) innervated the EDL in *Zfp106^−/−^* mice compared with 31 ± 0.4 motor units (*n* = 10) in WT littermates (*P* < 0.001). By 15 weeks, fewer motor units survived in *Zfp106^−/−^* mice, with 23 ± 1 motor units (*n* = 12) surviving compared with 31 ± 0.5 motor units (*n* = 10) in WT littermates (*P* < 0.001), suggesting a progressive loss of motor neurons.

Fast twitch muscles such as EDL normally fatigue rapidly and cannot maintain force when repeatedly stimulated. We therefore examined the fatigue characteristics of EDL muscles in 7- and 15-week-old *Zfp106^−/−^* mice, by undertaking a fatigue test in which the muscles were repeatedly stimulated (Fig. 2G and H). Typical fatigue traces from EDL muscles of WT and *Zfp106^−/−^* littermates are depicted in Figure 2H showing that in *Zfp106^−/−^* mice there was a clear shift in the fatigue pattern of EDL, becoming fatigue resistant as the mice aged. From traces such as these, a fatigue index (FI) was calculated for each muscle, where an FI of 1.0 indicates that a muscle is completely fatigue resistant, as previously described (13). Our findings revealed that 7-week-old WT mice had a FI of 0.32 ± 0.04 (*n* = 10) (Fig. 2G and H) compared with 0.48 ± 0.06 in 7-week-old *Zfp106^−/−^* (*n* = 12), an increase of 33.3%, although this difference was not statistically significant (*P* = 0.07). However, by 15 weeks of age, the FI of EDL in *Zfp106^−/−^* mice was 0.69 ± 0.05 (*n* = 12), compared with 0.23 ± 0.02 (*n* = 10) in WT littermates, a statistically significant increase of 66.7% (*P* < 0.001), and a statistically significant increase of 30.4% over 7-week-old *Zfp106^−/−^* mice (*P* = 0.019). These results show that in *Zfp106^−/−^* mice, EDL muscles are significantly more fatigue resistant than those of WT littermates, a characteristic usually observed in slow twitch muscles.

### *Zfp106 deficiency* causes progressive motor neuron degeneration with reactive gliosis and loss of motor and sensory axons

**Figure 3.**
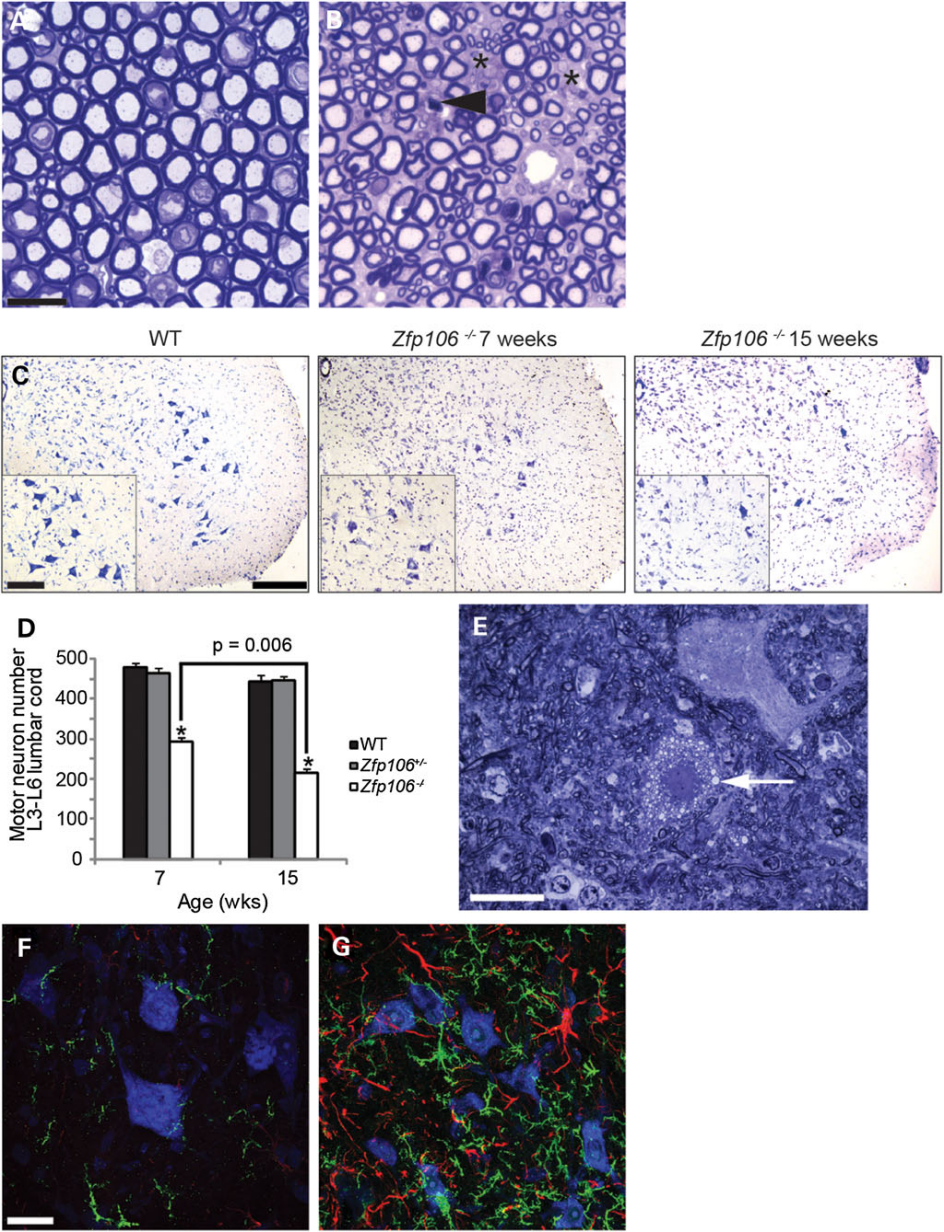
Motor neuron and axonal degeneration and gliosis in *Zfp106^−/−^* mouse spinal cord. (**A** and **B**) Representative toluidine blue stained sections of lumbar L4 and L5 ventral roots from 15-week old WT (A) and *Zfp106^−/−^* (B) mice. *Zfp106^−/−^* mice have fewer motor axons than WT littermates (spaces are marked with asterisks), and have degenerating axons, indicated with arrow head. Scale bar is 20 μm. (**C**) Representative images of the ventral horn of lumbar spinal cord sections stained for Nissl from WT, 7- and 15-week-old *Zfp106^−/−^* mice. The number of motor neurons in the sciatic pool (inset) was counted for each genotype and age to calculate motor neuron survival (**D**). Scale bars: main images 200 μm inset 100 μm. (D) The survival of motor neurons in the sciatic pool in female mice at 7- and 15-weeks of age. Motor neuron survival in 7-week-old *Zfp106^−/−^* mice (294 ± 11) is reduced compared with WT littermates (481 ± 9) and further reduced in 15-week-old *Zfp106^−/−^* mice (217 ± 9). Numbers represent the mean ± SEM. At least five animals were assessed per genotype per time point (*P < 0.001)*.* (**E**) Representative toluidine blue stained section of 15-week-old *Zfp106^−/−^* lumbar spinal cord showing an example of a vacuolated (arrow) motor neuron. (**F** and **G**) Lumbar spinal cord sections of 15-week-old WT (F) and *Zfp106^−/−^* (G) mice stained for IBA-1 (green), GFAP (red) and Nissl (blue). Immunoreactivity for micro- and astrogliosis is dramatically increased in the lumbar of 15-week-old *Zfp106^−/−^* mice compared with WT littermates. Scale bar is 20 μm.

Behavioural, physiological and histopathological studies of *Zfp106^−/−^* mice all indicate possible neuronal defects. Therefore, we examined the morphology of motor neurons and their axons from WT and *Zfp106^−/−^* littermates. Toluidine blue stained semi-thin sections from the lumbar L4 and L5 ventral roots revealed widespread loss of fibres, active degeneration of motor axons with no clear signs of regeneration and apparently normal myelination (Fig. 3A and B). To assess motor neuron survival, we counted the number of motor neurons from the sciatic pool in the lumbar spinal cord (L3-L6) of 7- and 15-week-old WT, *Zfp106^+/−^* and *Zfp106^−/−^* littermates (Fig. 3C and D). The number of motor neurons of 7-week-old *Zfp106^−/−^* mice is reduced by ∼35% compared with WT littermates and decreased by a further ∼15% at 15 weeks of age, corresponding to a total motor neuron loss of 50% in 15-week-old *Zfp106^−/−^* mice (Fig. 3C and D). Interestingly, visualization of toluidine blue stained semi-thin sections of the lumbar region of *Zfp106^−/−^* spinal cord showed the occasional presence of vacuolated motor neurons, likely to be undergoing cell death (Fig. 3E). Immunohistochemical staining of the lumbar spinal cord of 15-week-old WT and *Zfp106^−/−^* littermates using antibodies to GFAP (astrocytosis) and IBA-1 (microgliosis) also revealed widespread reactive gliosis, coincidental with over 50% motor neuron loss, not seen in WT or *Zfp106^+/−^* littermates (Fig. 3F and G, and data not shown).

We performed p62 staining of spinal cord sections from *Zfp106^−/−^* mice because p62 is commonly found localized into aggregates in neurodegenerative disease (14). However, we did not observe any altered localization or aggregation of p62 in the lumbar region of 15-week-old *Zfp106^−/−^* mice (data not shown). Terminal deoxynucleotidyl transferase dUTP nick end labelling (TUNEL) staining on ventral spinal cord of 15-week-old WT and *Zfp106^−/−^* mice showed cells undergoing active cell death in *Zfp106^−/−^* mice (Supplementary Material, Fig. S3A and B).

**Figure 4.**
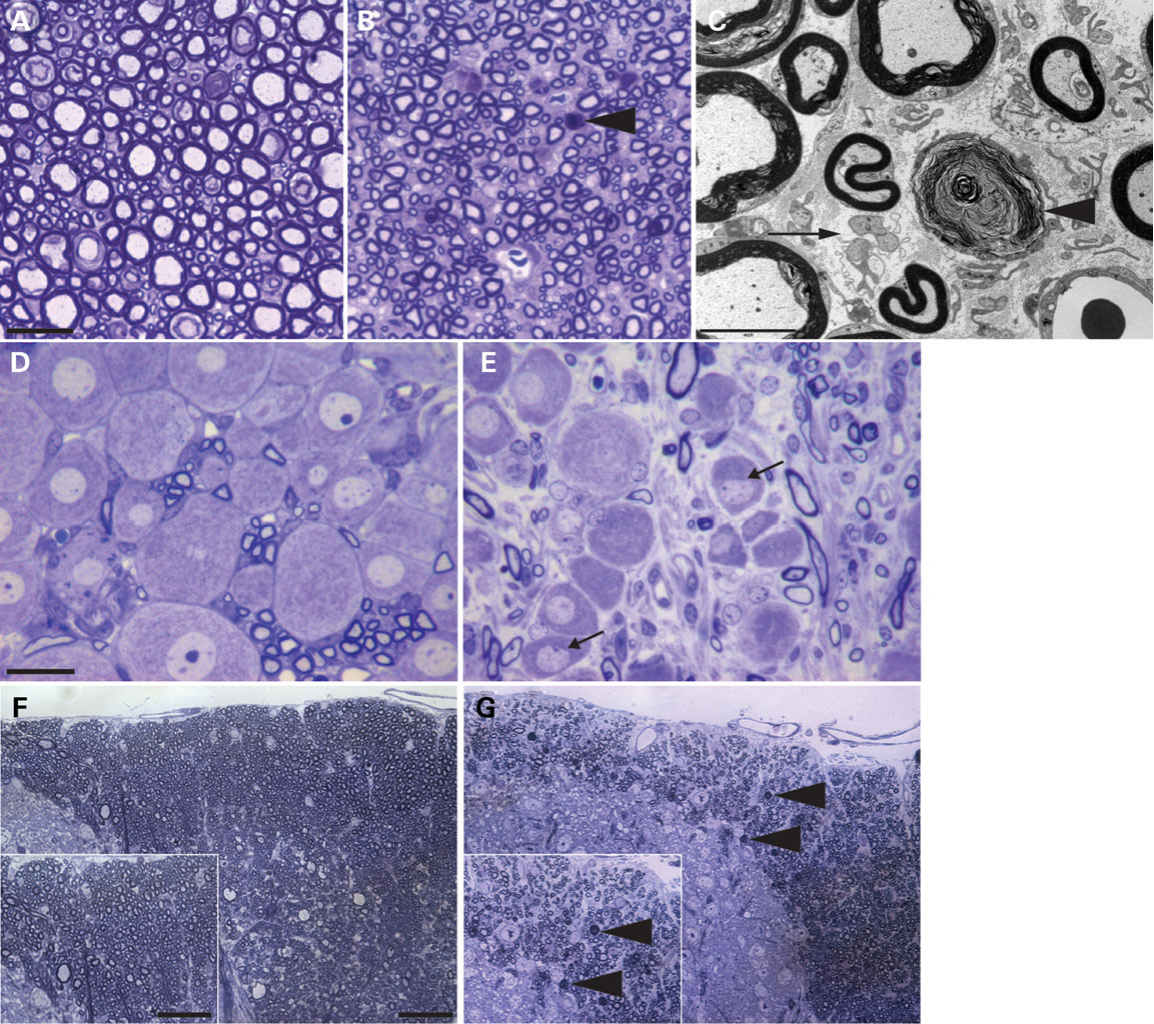
Axonal degeneration of sensory neurons. (A, B) Representative toluidine blue stained sections of lumbar L4 and L5 dorsal roots from WT (**A**) and *Zfp106^−/−^* (**B**) mice. *Zfp106^−/−^* mice have fewer sensory axons, and degenerating axons, indicated with arrow head. Scale bar is 20 μm. (**C**) Electron micrograph of *Zfp106^−/−^* dorsal root showing degenerating myelinated axons (arrow head) and evidence of loss of unmyelinated axons with denervated Schwann cells (arrow). Scale bar is 5 μm. (**D** and **E**) Representative toluidine blue stained section of lumbar DRG from WT (D) and *Zfp106^−/−^* (E) mice. Numerous DRG neurons from *Zfp106^−/−^* mice (E) are shrunken and irregular and some show signs of chromatolysis, with flattened nuclei (indicated with arrows). Scale bar is 20 μm. (**F** and **G**) Representative toluidine blue stained sections of the dorsal fasciculi from the lumbar spinal cord from WT (F) and *Zfp106^−/−^* (G) mice showing fewer axons and axonal degeneration (arrow heads). Scale bar is 20 μm. All images are of 15-week-old WT or *Zfp106^−/−^* mice, images representative of three animals per genotype.

Given the striking loss of lower motor neurons, we next examined the sensory system. Assessment of L4 and L5 dorsal roots from 15-week-old female WT and *Zfp106^−/−^* littermates revealed a loss of both large and small sensory axons (Fig. 4A and B). We also observed degeneration of myelinated and unmyelinated axons and no evidence of axonal regeneration or demyelination (Fig. 4C). In addition, numerous L4 and L5 dorsal root ganglia (DRG) of 15-week-old female *Zfp106^−/−^* mice were smaller compared with WT littermates, some showing signs of chromatolysis (Fig. 4D and E). Examination of dorsal fasciculi in the spinal cord revealed degeneration of ascending sensory axons (Fig. 4F and G). However, we did not observe any morphological or pathological defects in the descending spinal cord tracts (data not shown). Thus, *Zfp106^−/−^* mice have deficits in both the motor and sensory neuronal systems of the spinal cord.

### *Zfp106^−/−^* neuronal loss is not developmental

**Figure 5.**
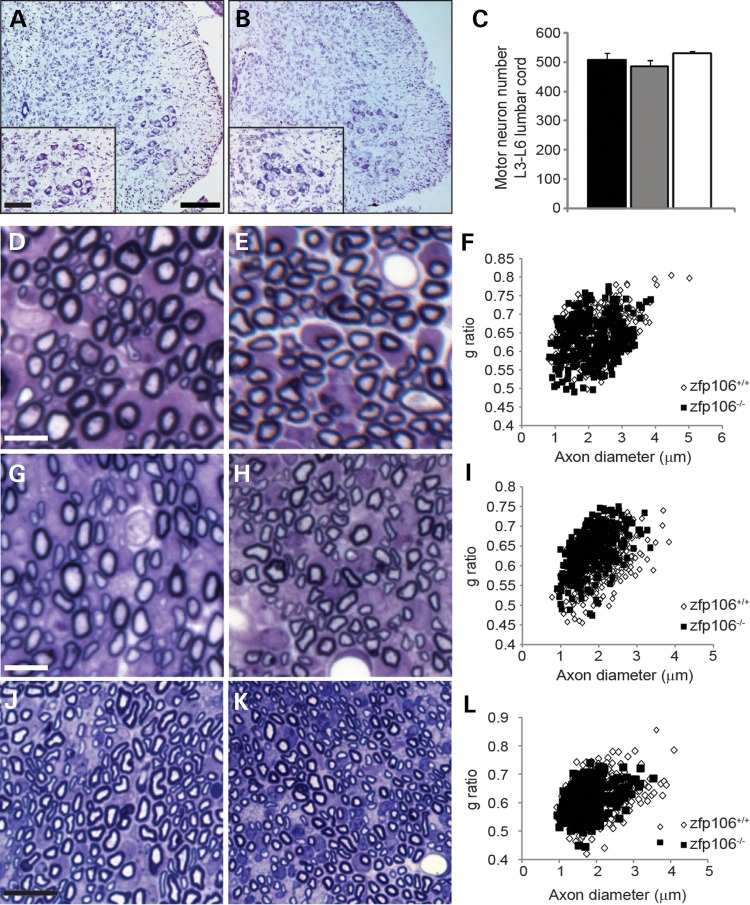
Neuronal survival and axonal morphology at P9. (**A** and **B**) Representative images of the ventral horn of lumbar spinal cord sections stained for Nissl from P9 WT and *Zfp106^−/−^* mice. The number of motor neurons in the sciatic pool (inset) was counted for each genotype. Scale bars: main images 200 μm, inset 100 μm. (**C**) Survival of motor neurons in the sciatic pool in female mice at P9. Motor neuron survival is unaffected in P9 *Zfp106^−/−^* mice (530 ± 15) compared with WT littermates (508 ± 23). Numbers represent the mean ± SEM. At least five animals were assessed per genotype per time point. (**D** and **E**) Representative toluidine blue stained sections of lumbar L4 and L5 ventral roots from P9 WT (D) and *Zfp106^−/−^* (E) mice. (**F**) Distribution graph relating g-ratio to axon diameter of all myelinated axons analysed from ventral roots show no differences between WT and *Zfp106^−/−^*. Scale bar is 20 μm. (**G** and **H**) Representative toluidine blue stained sections of lumbar L4 and L5 dorsal roots from P9 WT (G) and *Zfp106^−/−^* (H) mice. Scale bar is 20 μm. (**I**) Distribution graph relating g-ratio to axon diameter from dorsal roots. *Zfp106*^−/−^ mice have a significant shift in the frequency distribution towards a thinner axon diameter and an increased g-ratio compared with WT (*P* < 0.001). (**J** and **K**) Representative toluidine blue stained sections of the sciatic nerve from P9 WT (J) and *Zfp106^−/−^* (K) mice. Scale bar is 10 μm. (**L**) Distribution graph relating g-ratio to axon diameter for the sciatic nerve. *Zfp106*^−/−^ mice show a significant shift in the frequency distribution towards a smaller axon diameter compared with WT animals (*P* < 0.001) but no change in g-ratio.

Following the identification of progressive neurodegeneration in both motor and sensory neurons of the spinal cord, we next sought to establish whether neuronal abnormalities are present during development. Motor neuron survival was unaffected in P9 *Zfp106^−/−^* mice compared with WT littermates (Fig. 5A–C) and DRG from *Zfp106^−/−^* mice were also indistinguishable from WT littermates at this age (data not shown). Axonal morphology was quantified on toluidine blue stained semi-thin sections from the lumbar L4 and L5 ventral and dorsal roots, and from sciatic nerve sections of P9 animals (Fig. 5D–L and Supplementary Material, Fig. S4). Axonal morphology from ventral roots was similar between WT controls and *Zfp106^−/−^* mice (Fig. 5D–F). However, there was a significant reduction in axonal diameter on the sciatic nerve of *Zfp106^−/−^* mice when compared to WT controls (Fig. 5J–L and Supplementary Material, Fig. S4). This is accompanied with a significant shift in the frequency distribution towards a thinner axon diameter and an increased g-ratio in *Zfp106^−/−^* dorsal roots when compared with WT (Fig. 5G–I). Thus, deficiency of *Zfp106* leads to mild early changes in axonal morphology but does not cause neuronal loss at P9.

### *Zfp106* deficiency does not cause major abnormalities in the brain

We performed an immunohistological examination on the brains of female 15-week-old WT and *Zfp106^−/−^* littermates using the following stains and protein markers: H&E, GFAP, IBA-1, p62, ubiquitin, myelin basic protein and neurofilament. There were no major abnormalities in the anatomy of the brain or differences in reactive gliosis, p62, MBP or neurofilament staining between WT and *Zfp106^−/−^* mice (data not shown). Therefore, deficiency in *Zfp106* causes specific defects in the spinal cord and PNS but not the brain, despite being expressed at similar levels in the brain (Fig. 1B).

### Muscle histopathology in *Zfp106^−/−^* mice

**Figure 6.**
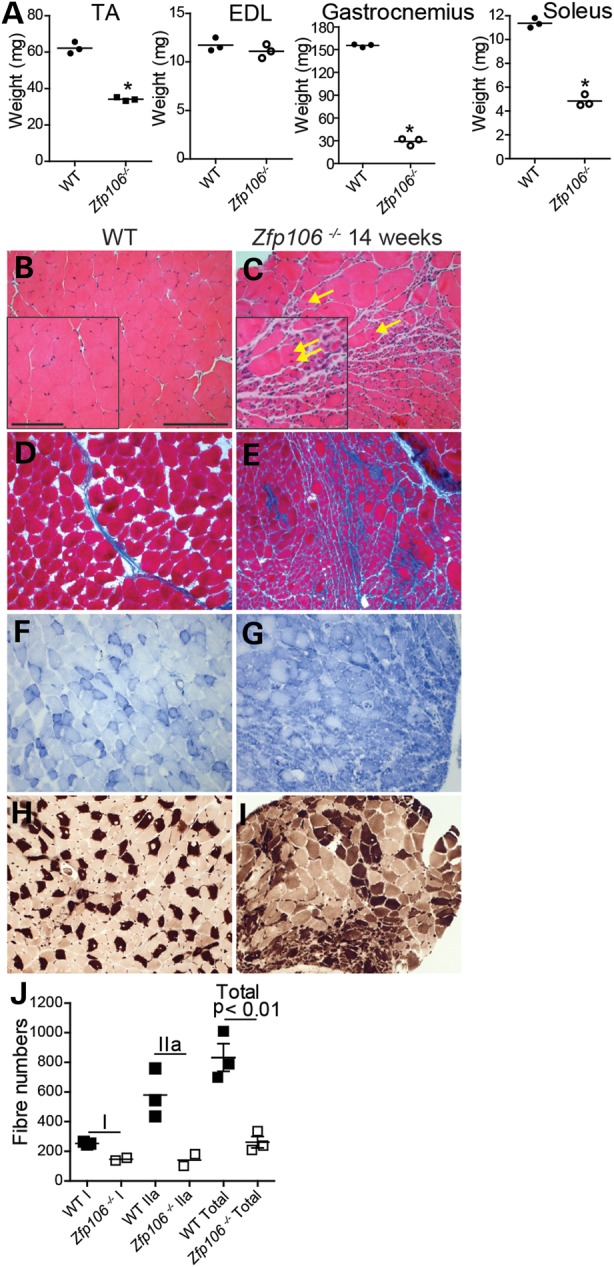
Muscle histology of WT and *Zfp106^−/−^* male mice. (**A**) Muscle weights for the TA (WT, 62 mg ± 1 mg; *Zfp106^−/−^*, 34 mg ± 1 mg), EDL (WT, 11 mg ± 0.4 mg; *Zfp106^−/−^*, 11 mg ± 0.4 mg), gastrocnemius (WT, 155 mg ± 1 mg; *Zfp106^−/−^*, 29 mg ± 23 mg) and soleus (WT, 11 mg ± 0.2 mg; *Zfp106^−/−^*, 5 mg ± 0.3 mg) from male 14-week old WT and *Zfp106^−/−^* littermates (*n* = 3). TA, gastrocnemius and soleus weights are significantly different from WTs (*P < 0.01). (**B–I**) Representative images of muscle histopathology from 14-week-old WT and *Zfp106^−/−^* mice: (B and C) H&E staining of the gastrocnemius shows significant atrophy, reduced fibre size and centralized nuclei (yellow arrows) in *Zfp106^−/−^* muscle compared with WT littermates; (D and E) Masson's trichome staining of gastrocnemius muscle reveals fibrosis in *Zfp106^−/−^* muscle (E), evident with increased blue staining; (F and G) NADH-TR staining of the gastrocnemius shows greatly increased dark blue staining in *Zfp106^−/−^* muscle (G) compared with WT littermates, indicative of a higher proportion of slower twitch, type I muscle fibres; (H and I) ATPase staining of *Zfp106^−/−^* soleus muscle (I) showing an increase in darkly stained fibres, indicating an increase in the proportion of type I fibres overall. Scale bars: main images 20 μm, inset 10 μm. (**J**) Type I (WT, 253 ± 6 fibres; *Zfp106^−/−^*, 147 ± 10 fibres), type IIa (WT, 579 ± 95 fibres; *Zfp106^−/−^*, 141 ± 40 fibres) and total fibre number (WT, 833 ± 92 fibres; *Zfp106^−/−^*, 261 ± 38 fibres) were counted for the soleus muscle from 14-week-old WT and *Zfp106^−/−^* littermates using ATPase staining. The soleus from *Zfp106^−/−^* mice has significantly fewer muscle fibres than that of WT mice (*P* < 0.01).

The changes in the physiological characteristics of the TA and EDL muscles of *Zfp106^−/−^* mice led us to perform a more in-depth muscle histopathological analysis. Examination of gross morphology of *Zfp106^−/−^* mice clearly showed a reduced mass and diminished size of muscles (Supplementary Material, Fig. S5A). Analysis of 6- and 14-week-old WT and *Zfp106^−/−^* littermates revealed reduced TA, gastrocnemius and soleus muscle weight in 14-week but not 6-week-old *Zfp106^−/−^* mice when compared with WT littermates (*P* < 0.01). However, no differences were found in EDL muscle weights at either 6 or 14 weeks of age (Fig. 6A and data not shown) or in tibia length from 14-week old WT and *Zfp106^−/−^* littermates (Supplementary Material, Fig. S5B). Histopathological analysis of muscles (gastrocnemius, soleus and EDL) from 6-week-old *Zfp106^−/−^* littermates was comparable to that seen in WT littermates (data not shown). However, Haematoxylin and eosin (H&E) staining of the gastrocnemius from 14-week-old *Zfp106^−/−^* mice, which had atrophied by ∼80% at that stage, revealed extensive muscle fibre atrophy, with areas of increased nuclearity, and evidence of reorganization and fibre regeneration (Fig. 6B and C). Extensive areas of the gastrocnemius were also fibrotic (Fig. 6D and E). We also assessed the distribution of fibre types within the gastrocnemius and soleus using NADH–tetrazolium reductase (NADH-TR) and ATPase stainings and found increased type I/highly oxidative fibres in the gastrocnemius and soleus of 14-week-old *Zfp106^−/−^* mice compared with WT littermates (Fig. 6F –I). Moreover, in all sections of soleus muscle from *Zfp106^−/−^* mice examined there was a greater spread of fibre size, ranging from very small to very large muscle fibres, compared with WT soleus muscle (Supplementary Material, Fig. S5C). In addition, the soleus of 14-week-old *Zfp106^−/−^* mice had fewer fibres when compared with WT littermates (Fig. 3J). Thus, *Zfp106* deficiency causes progressive muscle pathology.

### *Zfp106* deficiency affects genome-wide transcription

A role in transcriptional control has been recently proposed for ZFP106 (5). To assess how *Zfp106* deficiency might affect transcriptional regulation, we analysed genome-wide transcriptional changes from the spinal cords of 6-week-old *Zfp106^−/−^* mice using RNA-seq. At this stage, *Zfp106^−/−^* mice already display motor and sensory neuron pathology (Figs 3 and 4). Analysis of gene expression revealed a large number of differentially expressed genes, the majority of which were upregulated in *Zfp106*^−/−^ when compared with WT littermate spinal cords (Table 1 and Supplementary Material, Fig. S6A). Pathway analysis of differentially expressed genes showed a variety of affected cellular processes, prominently including the inflammatory response, metabolic disease, cell-to-cell signalling and interaction, hematological system function, immune cell trafficking and cell motility.

### *Zfp106* deficiency causes mitochondrial abnormalities

To investigate whether mitochondrial dysfunction may be a feature of *Zfp106^−/−^* neurons, we examined the mitochondrial properties of embryonic motor neurons derived from WT, *Zfp106^+/−^* and *Zfp106^−/−^* littermates.

**Figure 7.**
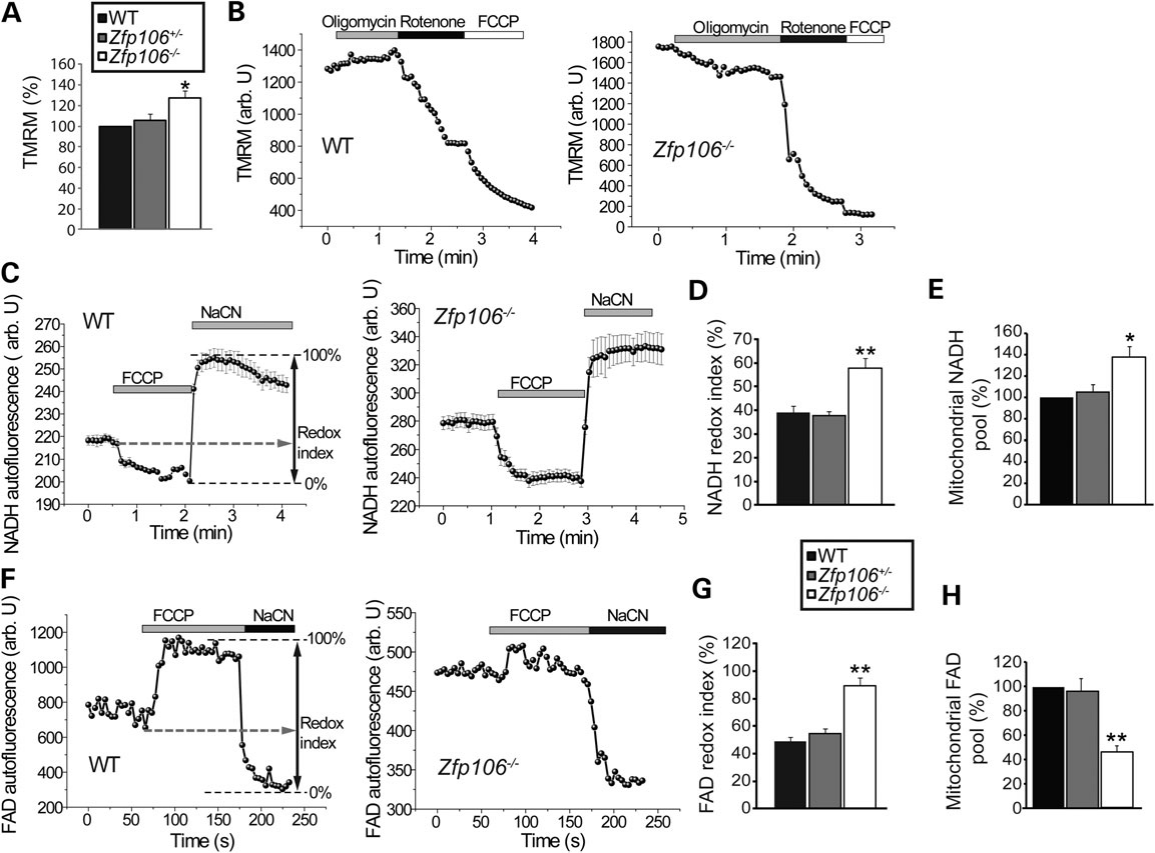
Mitochondrial abnormalities in *Zfp106^−/−^* embryonic motor neurons. (**A**) Resting Δ*ψ*_m_ from embryonic WT, *Zfp106^+/−^* and *Zfp106^−/−^* motor neurons, estimated by live cell imaging using TMRM. Data are normalized to resting Δ*ψ*_m_ for WT (100%) and represent the mean ± SEM. (**B**) Representative traces of Δ*ψ*_m_ (TMRM, arbitrary units) in response to oligomycin (2 μg/ml) (inhibitor of the F_1_F_O_-ATP synthase), rotenone (5 μm) (inhibitor of complex I) and the minimum TMRM fluorescence achieved after addition of FCCP 1 μm (uncoupling agent) for WT and *Zfp106^−/−^* embryonic motor neurons. Following oligomycin addition WT motor neuron Δ*ψ*_m_ hyperpolarises, whilst *Zfp106^−/−^* depolarises. (**C**) NADH autofluorescence monitored in WT and *Zfp106^−/−^* motor neurons, the addition of FCCP maximizes mitochondrial respiration, thus minimizes mitochondrial NADH. NaCN was added to block mitochondrial respiration and therefore maximize mitochondrial NADH. Redox index (the initial redox level expressed as a percentage of the range) and mitochondrial NADH level (the difference in absolute values between the minimum and maximum NADH autofluorescence) are described graphically. (**D**) NADH redox index for WT, *Zfp106^+/−^* and *Zfp106^−/−^* motor neurons calculated from (C). NADH redox index is increased in *Zfp106^−/−^* embryonic motor neurons (58% ± 4%) compared with WT littermates (39% ± 3%). (**E**) Mitochondrial pool of NADH represented as % of WT. NADH levels are increased in *Zfp106^−/−^* (138% ± 9.1%) motor neurons when compared with WT. (**F**) FAD autofluorescence monitored in WT and *Zfp106^−/−^* motor neurons, the addition of FCCP maximizes respiration, increasing FAD autofluorescence to maximal levels, whilst NaCN inhibits respiration, reducing FAD autofluorescence to minimal levels. (**G**) FAD redox index for WT, *Zfp106^+/−^* and *Zfp106^−/−^* motor neurons, calculated by normalizing the FCCP response to 100% and the NaCN response to 0%. FADH redox index is increased in *Zfp106^−/−^* embryonic motor neurons (89% ± 6%) compared with WT littermates (49% ± 3%). (**H**) Mitochondrial pool of FAD represented as % of WT. FAD levels are decreased in *Zfp106^−/−^* motor neurons (45.6% ± 5.2%) when compared with WT. Data from (A–H) are represented as mean ± SEM; *n* ≥ 34 motor neurons per genotype, per condition. *P < 0.05; **P < 0.01.

We first assessed mitochondrial membrane potential (Δ*ψ*_m_), an indicator of the mitochondrial energetic state, measured using tetramethylrhodamine methylester (TMRM), a cell-permeable, potentiometric, fluorescent dye that is readily sequestered by active mitochondria (15,16). TMRM is a positively charged molecule which accumulates within mitochondria in inverse proportion to Δ*ψ*_m_; hyperpolarized mitochondria will accumulate more dye, and hypopolarized mitochondria will accumulate less dye. Fluorescent dye accumulation in mitochondria is then optically detected by confocal microscopy. We found that mitochondria in motor neurons derived from *Zfp106^−/−^* mice were associated with a significant increase (27% ± 7%, *P* < 0.001) in the TMRM signal compared with mitochondria from WT motor neurons (*n* = 34 cells) (Fig. 7A). In *Zfp106^+/−^* motor neurons, values of TMRM fluorescence were not significantly different from WT (Fig. 7A). These data suggest that *Zfp106* deficiency is associated with increased basal Δ*ψ*_m_ in embryonic motor neurons, at least *in vitro.*

We next investigated how *Zfp106* deficiency results in an increase in Δ*ψ*_m_ in embryonic motor neurons from *Zfp106^−/−^* mice when compared with *Zfp106^+/−^* and WT littermates. In cells with normal oxidative phosphorylation, Δ*ψ*_m_ is maintained by the proton pump activity of the respiratory chain. However, if oxidative phosphorylation is impaired, then hydrolysis of ATP by the F_1_F_O_-ATP synthase (Complex V) can pump protons across the inner membrane and so maintain Δ*ψ*_m_ (17). Application of the inhibitor of the F_1_F_O_-ATP synthase, oligomycin, to WT motor neurons increased Δ*ψ*_m_ by 3 ± 0.1% (*n* = 23) (Fig. 7B), and in *Zfp106^+/−^* motor neurons by 5 ± 0.3% (*n* = 27). However, exposure of *Zfp106^−/−^* motor neurons to oligomycin caused a profound decrease in Δ*ψ*_m_ (TMRM signal fell by 17% ± 3%, *P* < 0.001, *n* = 32) (Fig. 7B). Thus, in *Zfp106^−/−^* motor neurons, the impaired activity of the respiratory chain switches the F_1_F_O_ complex to an ATP consumption mode which serves to maintain Δ*ψ*_m_.

The redox state of NADH or FAD is a function of respiratory chain activity and the rate of substrate supply. We measured the resting level of NADH and FAD^++^ autofluorescence and generated the ‘redox index’; a ratio of the maximally oxidized (response to FCCP) and maximally reduced (response to sodium cyanide (NaCN)) signals. The redox index of NADH in *Zfp106^−/−^* motor neurons was significantly more oxidized than in WT littermates (WT: 39% ± 3%, *n* = 35 versus *Zfp106^−/−^*: 58% ± 4%, *n* = 46; *P* < 0.001) (Fig. 7C and D). We also estimated the levels of the mitochondrial pool of NADH, the substrate for complex I. NADH levels were significantly increased in *Zfp106^−/−^* motor neurons (Fig. 7E) when compared with WT controls. Thus, the combination of accumulating substrate and a diminished redox index in *Zfp106^−/−^* motor neurons suggest that *Zfp106* deficiency leads to functional inhibition of the NADH-dependent (Complex I) mitochondrial respiration.

In addition, FAD^++^ levels were increased in *Zfp106^−/−^* motor neurons compared with WT littermates (WT: 49% ± 3% versus *Zfp106^−/−^* 89% ± 6%; *n* = 47; *P* < 0.001) (Fig. 7F and G). The levels of the mitochondrial pool of FAD were also estimated; FAD levels were significantly lower in *Zfp106^−/−^* motor neurons when compared with WT controls (Fig. 7H). These findings suggest that *Zfp106* deficiency results in activation of Complex II respiration. The FAD redox state in WT motor neurons (*n* = 37) was indistinguishable from *Zfp106^+/−^* motor neurons (*n* = 44) (Fig. 7F and G). Thus, the striking abnormalities in motor neuron mitochondrial properties may underlie the neurodegenerative effects caused by *Zfp106* deficiency.

## Discussion

*Zfp106* is expressed during development and ubiquitously in adulthood, however, its functions remain unknown. Here, we report a new *Zfp106*-deficient mouse strain, which presents with reduced body weight from 3 weeks of age, a reduction in grip strength, the appearance of progressive gait abnormalities and the development of a severe kyphosis compared with WT and *Zfp106^+/−^* littermates (Fig. 1 and Supplementary Material, Movie S1). These phenotypes were associated with neuromuscular deficits. Physiological and histopathological analyses revealed that *Zfp106^−/−^* mice undergo a progressive loss of motor neurons, degeneration of motor and sensory axons together with progressive, severe muscle atrophy (Figs 2, 3, 4 and 6). Importantly, we established that neurodegeneration is progressive and does not occur during development, as motor and sensory neurons were intact at P9. However, mild changes in axonal morphology were present at this early stage (Fig. 5), suggesting that the neurodegeneration process may be already starting.

Interestingly, despite Z*fp106* being expressed throughout the CNS (2), abnormalities in *Zfp106^−/−^* mice are confined to lower motor neurons, sensory neurons and their peripheral axons, and in the dorsal spinal cord ascending tracts (Figs 3 and 4). No major morphological or pathological abnormalities were observed in the descending spinal cord tracts or in other brain regions such as cerebellum, basal ganglia, thalamus, hippocampus or cortex. Thus, preservation of *Zfp106* expression levels is essential for the maintenance and survival of spinal cord residing neurons and their axons, suggesting that spinal cord residing neurons, with their exceptionally long axons, are particularly susceptible to changes in *Zfp106* expression. It may be possible that neurons from other brain areas may also be compromised, but the rapid degeneration of spinal cord neurons may preclude pathology potentially appearing in other areas. Future studies conditionally deleting *Zfp106* specifically in different brain areas will be required to address this issue.

Histopathological assessment of young (6-week-old) *Zfp106^−/−^* muscle did not reveal overt pathological abnormalities (data not shown), whilst 14-week-old *Zfp106^−/−^* mice display varying degrees of muscle involvement ranging from no significant atrophy in the EDL to an ∼80% weight loss of the gastrocnemius (Fig. 6). This suggests that the observed muscle pathology may be secondary to the neuronal degeneration. However, it is possible that deficiency of *Zfp106* in muscle also contributes to the neurodegeneration, and future studies that conditionally remove *Zfp106* from neurons or muscle will clarify this point.

In humans, ZFP106 is localized to the nucleus, and with the presence of N- and C-terminal zinc finger motifs, is likely to regulate gene expression (1). Although several commercial antibodies are available against human ZFP106, none of them recognize endogenous mouse ZFP106, at least from brain and spinal cord (data not shown). Thus, we used RNA-seq analysis to gain insight into the functional consequences of *Zfp106* deficiency, showing a number of genes misregulated due to *Zfp106* deficiency. Pathway analysis of the differentially regulated genes supports a role for *Zfp106* in neuronal maintenance and survival. Previous research has shown that NRF-1, a transcription factor linked to the regulation of mitochondrial function (18) and neurite outgrowth (19), is important in the transcription of full length *ZFP106* (2). *Nrf1* null mice are embryonic lethal, however, mice in which *Nrf1* is specifically deleted from the CNS (CNS conditional *Nrf1^−/−^* mice) develop a juvenile aggressive neurodegenerative phenotype (20,21). Neurodegeneration is more widespread in CNS conditional *Nrf1^−/−^* mice compared with *Zfp106^−/−^* mice, involving alterations in the hippocampus and cerebellum but, importantly, CNS conditional *Nrf1^−/−^* mice display pathology in the ventral horn of the spinal cord, including increased oxidative stress. Whether *Zfp106* plays a role in the development of the *Nrf1^−/−^* conditional knock out phenotype remains uncertain, however, the effect of *Nrf1^−/−^* on spinal cord neuronal morphology has similarities to the abnormalities observed here in *Zfp106^−/−^* mice. The contribution of ZFP106 to neuronal survival within the spinal cord and PNS may suggest a specific role as a downstream regulator of NRF-1.

Mitochondrial dysfunction has been proposed to be an instigating factor in many neurodegenerative conditions (22). We show here that a deficiency in *Zfp106* causes profound mitochondrial abnormalities in embryonic motor neurons (Fig. 7). Deficiency of *Zfp106* induces inhibition of Complex I within the electron transport chain of mitochondria and activation of Complex II. It has been previously shown that Complex II can become activated in order to compensate for Complex I inhibition, leading to the hydrolysis of ATP by the F_1_F_O_-ATP synthase (Complex V) and the pumping of protons across the inner mitochondrial membrane (23,24). Overall, these abnormalities result in an increase in Δ*ψ*_m_, as observed in *Zfp106^−/−^* embryonic motor neurons. Interestingly, elevated Δ*ψ*_m_ can lead to an increase in the production of reactive oxygen species (ROS) and can be a cause of neuronal cell death (25,26). Furthermore, inhibition of Complex I and the resulting activation of Complex II have been shown to reverse the flow of electrons within the electron transport chain and produce free radicals (27), which can in turn lead to mitochondrial and cellular damage. Although we provide strong evidence of mitochondrial dysfunction due to *Zfp106* deficiency, whether this dysfunction is directly or indirectly mediated by ZFP106 remains an open question.

Mitochondrial abnormalities have been reported in a variety of neurodegenerative disorders affecting motor and sensory neurons in the spinal cord and their axons, including motor neuron diseases such ALS (28–30), spinal muscular atrophy (SMA) (31) and prominently in peripheral neuropathies, including Charcot-Marie-Tooth (CMT) (32). A number of causative CMT genes are associated with mitochondrial function, including: *MFN2*, *GDAP1* or MT-ATP6 (33–35) and the Δ*ψ*_m_ of CMT patients carrying mutations in *MFN2* or *GDAP1* is disrupted (36,37). Furthermore, peripheral neuropathy is observed in patients with mitochondrial diseases, such as Leigh's disease, mitochondrial neurogastrointestinal encephalopathy (MNGIE), neuropathy, ataxia and retinitis pigmentosa (NARP) (38) and spastic paraplegia with optic atrophy and neuropathy (SPG55) (39).

Any possible role for ZFP106 in human disease remains to be established, as disease-causing mutations in *ZFN106* have not yet been identified. The neuro-anatomical phenotypes observed in *Zfp106^−/−^* mice have striking similarities with human axonal sensory-motor neuropathies, such as CMT type 2 (CMT2). CMT2 prevalently affect the sensory and motor nerves, but also show loss of MNs, spinal cord gliosis and degeneration of the fasciculus gracilis, as documented here for *Zfp106*^−/−^ mice. Recessive forms of CMT2 usually have an earlier onset, occurring in the first decade of life and frequently before the age of two (40,41). These characteristics are similar to the deficits observed in *Zfp106^−/−^* mice, which start developing symptoms at weaning (Fig. 1 and Supplementary Material, Movie S1). However, since the function of human ZFP106 is not yet clear, it will be important to screen for *ZFN106* mutations in early-onset CMT2 cases (42), but also milder forms of CMT, as well as other neurodegenerative disease in which there is degeneration of either motor or sensory neurons, such as ALS and progressive muscular atrophy (PMA).

*ZFN106* is located on human chromosome 15 within the critical region of a previously mapped familial juvenile ALS linked region (ALS5) (43). Although a recent report suggests that mutations in Spatacsin (*SPG11*) are causative of ALS5, it is not clear if *SPG11* mutations explain all available ALS5-linked kindreds (43–45). Interestingly, a recent report identified ZFP106 as a novel factor regulating the initiation of transcription by targeting RNA-polymerase I to the promoter region of ribosomal RNA genes (5). This, together with ZFP106 proposed localization to the nucleolus links ZFP106 function with nucleolus function and RNA metabolism abnormalities, which together with mitochondrial dysfunction, are three proposed common pathomechanisms leading to ALS (46,47).

In conclusion, our findings indicate that *Zfp106* deficiency impairs mitochondrial function and is required for the maintenance and survival of specific motor and sensory neurons *in vivo*. Although no *ZFN106* mutations have yet been found in human disease, the phenotype of *Zfp106^−/−^* mice, comprising spinal cord specific neuronal deficits and mitochondrial dysfunction, raises the intriguing and novel possibility that ZFP106 is involved in neurodegenerative disease.

## Materials and Methods

### Mice

*Zfp106^−/−^* mice (*Zfp106*^tm1a(KOMP)Wtsi^) were generated through the Knockout Mouse Project (KOMP), which aims to produce embryonic stem cells with knockouts in a number of mouse genes all of which will be publically available (http://www.komp.org/geneinfo.php?geneid=87357). The *Zfp106^−/−^* mice were generated by targeting C57BL/6N ES cells using a knockout first, promoter-less allele (tm1a(KOMP)Wtsi) (http://www.komp.org/alleles.php#conditional-promoterless-csd). The construct targeted *Zfp106* intron 1, inserting a Neomycin resistance and a *LacZ* reporter cassette flanked by flippase recombinase (Flp) target sites (FRT). LoxP sites were also engineered flanking *Zfp106* exon 2, allowing for the conversion to a conditional allele via Flp recombination, followed by crossing to appropriate Cre lines. We confirmed the location of the tm1a(KOMP)Wtsi allele in the *Zfp106* gene of *Zfp106^−/−^* mice using Southern blotting (data not shown).

WT, *Zfp106^+/−^* and *Zfp106^−/−^* mice were on a hybrid C57BL/6NTac/Den;C57BL/6J-Tyrc-Brd;C57BL/6N background and assessed as littermates. Experiments were performed blind to genotype and the humane endpoint was defined as a loss of 20% body weight or an inability to right after 20 s. Animals were assessed daily and weighed weekly.

### Behavioural analysis

WT, *Zfp106^+/−^* and *Zfp106^−/−^* littermate female only mice were assessed for grip strength (BioSeb, Chaville, France) of all four limbs, as per the manufacturer's instructions. Grip strength readings were taken twice each at 6- and 13-weeks of age and the mean of both values for each time point per animal was used. Cohort sizes per time point were as follows: 6-weeks: 16 WT, 16 *Zfp106^+/−^* and 14 *Zfp106^−/−^* animals; 13-weeks: 13 WT, 9 *Zfp106^+/−^* and 7 *Zfp106^−/−^* animals.

WT, *Zfp106^+/−^* and *Zfp106^−/−^* littermate female mice were placed on an accelerating rotarod (Ugo Basile) twice a day, three times a week, at 6- and 12-week time points, as described previously (48). Cohort sizes per time point were as follows: 7-weeks: 6 WT, 10 *Zfp106^+/−^* and 14 *Zfp106^−/−^* animals; 12-weeks: 7 WT, 8 *Zfp106^+/−^* and 8 *Zfp106^−/−^* animals.

Modified SHIRPA methods were performed as described previously (10,11). Tremors were qualitatively assessed by observation in a viewing jar and recorded as having tremors (1) or not (0). Limb grasping was assessed as either clasping one or more limbs (1) or not (0). The wire manoeuvre was assessed by hanging animals from a wire by their forelimbs and recording the degree to which they were able to hold their body weight. Animals were only scored as defective if they could not hang onto the wire for more than 1 s. Negative geotaxis involves animals walking down a vertical grate. Animals that slipped down at least half of the wire grate were scored as defective. Mice were subject to modified SHIRPA analysis at 6-weeks of age and included: 15 WT, 15 *Zfp106^+/−^* and 17 *Zfp106^−/−^* animals.

WT, *Zfp106^+/−^* and *Zfp106^−/−^* littermate female mice were monitored in an open field paradigm (Noldus Ethovision 3.1). Animals were placed in an arena and monitored using Ethovision 3.1 tracking software for 30 min at 7- and 14-week time points. The parameters distance moved (cm) and velocity (cm/s) were averaged per genotype and time point. Cohort sizes per time point were as follows: 7 weeks: 15 WT, 13 *Zfp106^+/−^* and 14 *Zfp106^−/−^* animals; and 14 weeks: 5 WT, 9 *Zfp106^+/−^* and 6 *Zfp106^−/−^* animals.

WT, *Zfp106^+/−^* and *Zfp106^−/−^* littermate female mice accuracy of paw placement was analysed using Locotronic® (Intelli-Bio, France). Briefly, animals begin in a starting area, illuminated with white light and move down a corridor consisting of a flat ladder (bars: 3 mm diameter, spaced by 7 mm) with three infrared sensors above and below each bar space, which registered movement errors 100 times a second. Animals were assessed three times each at 7- and 14-weeks of age; averages were taken per animal and at each time point. Cohort sizes per time point were as follows: 7 weeks: 10 WT, 12 *Zfp106^+/−^* and 14 *Zfp106^−/−^* animals; and 14 weeks: 6 WT, 7 *Zfp106^+/−^* and 5 *Zfp106^−/−^* animals.

### Physiological assessment of hindlimb muscle force, motor units and FI

Muscle force, motor unit number and FI for female WT, *Zfp106^+/−^* and *Zfp106^−/−^* littermates were examined at 7- and 15-weeks of age, as described previously (49). Cohort sizes per time point were as follows: 7 weeks: 5 WT, 6 *Zfp106^+/−^* and 6 *Zfp106^−/−^* animals; and 15 weeks: 5 WT, 5 *Zfp106^+/−^* and 6 *Zfp106^−/−^* animals. Briefly, mice were anesthetized (4.5% chlorohydrate, 1 ml/100 g of body weight), the distal tendons of the TA and EDL muscles in both hindlimbs attached to isometric force transducers (Dynamometer UFI Devices, Welwyn Garden City, UK), and the sciatic nerve exposed and sectioned. Isometric contractions were elicited by stimulating the nerve to the TA or EDL using squarewave pulses of 0.02 ms duration at supra-maximal intensity, via silver wire electrodes. Contractions were elicited by trains of stimuli at frequencies of 40, 80 and 100 Hz and measured using Scope software. The number of motor units innervating the EDL was determined by stimulating the motor nerve with stimuli increasing intensity, resulting in incremental increases in twitch tension due to successive recruitment of motor axons. The number of increments was counted giving an estimate of the number of functional motor units present in EDL muscle. The FI for the EDL muscle was examined by stimulating the EDL at 40 Hz for 250 ms every second for 180 s. Contractions were recorded with a pen recorder (Lectromed Multitrace 2) and the FI calculated as a measure of muscle fatigability and expressed as a ratio: FI = *F*_t180_/*F*_t0_.

### Morphological assessment of muscle and neuronal tissue

Ten micrometer cross-sections of frozen gastrocnemius, soleus and EDL were examined with H&E, Masson's trichrome and NADH-TR activity to determine the oxidative capacity of the muscle fibres, as described previously (50). Then, 10 μm cross-sections of the soleus were subjected to ATPase staining (acid preincubation at pH 4.47) (51) and the total number of fibres and proportion of fibre types within the soleus were assessed (52). Muscle morphology was analysed in WT and *Zfp106^−/−^* littermates at 6- and 14-weeks of age (male, *n* = 3 per genotype).

For assessment of motor neuron survival, the animals were terminally anaesthetized with 4.5% chlorohydrate (1 ml/100 g of body weight), transcardially perfused with 4% PFA and the lumbar region of the spinal cord removed, post-fixed in 4% PFA and cryopreserved in 30% sucrose. Serial 20 μm transverse sections were cut on a cryostat, stained with gallocyanin, a Nissl stain (53) and the number of positively stained motor neurons in the sciatic motor pool of every third section between the L3 and L6 levels of the spinal cord was counted, as previously described (12,13). Only large, polygonal neurons with a clearly identifiable nucleus and nucleolus were included in counts. This protocol avoids the possibility of counting the same cells twice in consecutive sections. A total of 40 sections were examined per spinal cord and at least five female mice were analysed from each experimental group.

For analysis of ventral and dorsal roots, DRG, and corticospinal tracts animals were perfused transcardially with saline followed with 3% glutaraldehyde in 0.05 M sodium cacodylate. Right L4 and L5 DRG with the attached dorsal and ventral roots, and spinal cords were dissected, post-fixed in 1% osmium tetroxide (Agar Scientific) and processed into araldite CY212 epoxy resin (Agar Scientific) through graded alcohols and propylene oxide using a standard protocol. Semi-thin sections (0.8 μm) were cut on an Ultracut E ultra-microtome (Leica), stained with 1% toluidine blue containing 1% borax (BDH), and examined with a Dialux light microscope. Neuronal morphology was analysed from 15-week-old WT and *Zfp106^−/−^* littermates (female, *n* = 3 per genotype).

### Axon morphology

Images of three semi-thin sections at P9 from lumbar L4 and L5 dorsal and ventral roots and sciatic nerve sections were selected randomly per animal. Three animals per genotype were used. To calculate axon diameter and g-ratios of myelinated axons, three random regions from each picture were analysed. All myelinated axons within each region were measured to calculate the g-ratio and axon diameter. In total, approximately 40 axons within each section and more than 120 axons per animal were analysed using AxioVision LE Rel. 4.2 software. The examiner was blind to genotype.

### Electron microscopy

WT and *Zfp106^−/−^* littermates were perfused as above (3% glutaraldehyde in 0.05 M sodium cacodylate) and dorsal roots dissected. Ultrathin sections 80 nm thick were cut from selected areas of the tissue, stained with uranyl acetate and lead citrate, and examined at 80 kV in a Philips CM10 electron microscope. Images were taken using a Megaview 3 digital camera and Olympus iTEM software. Morphology was analysed from 15-week-old WT and *Zfp106^−/−^* littermates (female, *n* = 3 per genotype).

### Immunocytochemistry

For the analysis of brain histopathology 15-week-old WT and *Zfp106^−/−^* mice were perfused transcardially with saline followed with 4% PFA, brain transferred to formalin and embedded in paraffin wax. Staining with H&E and anti- GFAP, IBA-1, p62, MBP and neurofilament antibodies was undertaken using a Ventana automated immunohistochemical staining machine (Ventana Medical Systems, Tuscon, AZ, USA). Paraffin-embedded sections once incubated with appropriate secondary antibodies were developed using 3,30-diaminobenzidine and counterstained with haematoxylin.

Twenty micrometer transverse sections from the lumbar region of fixed spinal cords collected onto glass slides were immuno-fluorescently stained with anti-GFAP (Cy-conjugated mouse monoclonal, Sigma; used at 1:1000) and anti-IBA1 (rabbit polyclonal, Abcam; used at 1:500), visualized using a secondary AlexaFluor488-conjugated antibody (Invitrogen) and counterstained with NeuroTrace® 435⁄455 fluorescent Nissl stain (Invitrogen). A C-terminal p62 antibody (Progen, Germany; 1:300) was also used to stain lumbar spinal cord sections. Confocal images were taken using a Zeiss 710 microscope. Active cell death was analysed using TUNEL staining (Promega) following manufacturer recommendations.

### Immunoblot

Fresh frozen spinal cords from female mice at 6 and 16 weeks of age were homogenized in RIPA buffer with protease inhibitors (complete mini EDTA-free, Roche). After centrifuging the homogenate at 12 000×*g* at 4°, the supernatant was used for western-blot analysis using pre-cast SDS gels (Invitrogen). The primary antibodies assessed included rabbit anti-actin (Sigma), LC3II (Nanotools) and ZFP106 (Novus, Santa Cruz, Bethyl). The Odyssey infrared imaging system (Li-Cor) was used for band detection and quantification.

### RNA-seq

Whole spinal cords from male, 6-week old *Zfp106^tm1a(KOMP)Wtsi^* mice (WT, *n* = 6; *Zfp106^−/−^*, *n* = 5) were homogenized and total RNA extracted using an RNeasy fibrous tissue extraction kit (Qiagen). RNA integrity was confirmed and concentration measured (∼0.2 mg/ml) using a Bioanalyzer (Agilent). cDNA libraries were created from 5 μg total RNA as follows. Using a TruSeq RNA Sample Prep v2 kit (Illumina), poly-A tailed RNA (mRNA) was purified from total RNA using an oligo dT magnetic bead pull-down. The resulting mRNA was fragmented using metal ion-catalysed hydrolysis and random priming used to synthesize double-stranded cDNA. End repair was performed with a combination of fill-in reactions and exonuclease activity to produce blunt ends. A single ‘A’ base was added to blunt ends followed by ligation to Illumina Paired-End Sequencing adapters containing unique index sequences, allowing samples to be pooled. The resulting libraries were amplified through 10 cycles of PCR using KAPA Hifi Polymerase, products were pooled based on a post-PCR Agilent Bioanalyzer, then the pool was size-selected using the LabChip XT Caliper (200–300 bp range). The multiplexed library was sequenced on the Illumina HiSeq 2000 (75 bp paired-end read length) aiming for >3 Gigabases of data per sample. Sequencing reads were aligned against the NCBI build 37.2 of the *Mus musculus* reference genome using the software TopHat (54). The TopHat alignment incorporated the reference genes annotations (GTF format) provided as part of the Illumina iGenomes project. Duplicate reads were filtered using the PICARD tools. Read counts per gene and per exon were computed using the python scripts provided with the DESeq/DEXSeq packages. Differential expression at the gene level was computed using the R package DESeq (55) and at the exon level using the R package DEXSeq (56).

### qPCR

Gene expression of *Zfp106* from 6-week-old WT and *Zfp106^−/−^* mice (female, *n* = 3 per genotype): ∼20–25 mg of brain or spinal cord was homogenized and RNA extracted using a Qiagen RNeasy fibrous tissue extraction kit. 200 ng of total RNA was used in a 10 μl reaction using the TaqMan RNA-to-CT One Step kit (Applied Biosystems). A TaqMan probe flanking the *Zfp106* splice acceptor region was used with GAPDH as an endogenous control (Applied Biosystems) in a multiplex primer-limited reaction. Reactions were performed in triplicate on an ABI Viia7 qPCR machine and analysed with Viia7 1.1 software. Gene expression data for *Zfp106^−/−^* tissues were analysed using the ΔΔCT method and normalized using GAPDH as endogenous reference gene (which expression is not changed by Zfp106 deficiency via RNA-seq, data not shown) relative to WT tissues (57).

### Northern blot

Total mRNA was isolated from brains and spinal cords using the Qiagen RNeasy lipid kit. RNA concentration was estimated using Nanodrop (Thermo scientific). 3′UTR probes were cloned from WT brain or spinal cord genomic DNA into a TOPO-TA (Invitrogen) vector by PCR. Digoxigenin-labeled antisense RNA 3′UTR probes were generated through *in vitro* transcription (Roche). RNA and an RNA ladder (Invitrogen) were run on formaldehyde gels (Ambion) for 2–3 h, then transferred upright for 4 h to N+ nylon membrane. RNA was crosslinked with a stratalinker, then the membrane was pre-hybridized with Ambion hybridization solution at 68°C. Probe was denatured and added to hybridization solution, and hybridization was carried out overnight. After membrane washings, CDP-Star development solution was used to detect bound probe. Membranes were exposed to X-ray film and developed.

### Embryonic motor neuron culture

Motor neuron cultures were prepared from WT, *Zfp106^+/−^* and *Zfp106^−/−^* littermate embryos as previously described (58). Briefly, embryonic spinal cords (E13.5) were removed, ventral horns isolated, motor neurons plated at 40 000 cells/cm^2^ onto cover-slips; cover-slips were coated with 1.5 mg/ml polyornithine (Sigma Aldrich) in sterile water for a minimum of 2 h followed with 1 mg/ml laminin (Sigma Aldrich) in L-15 medium (Sigma Aldrich) for 2 h. Motor neurons were maintained in complete neurobasal medium (CNB); 200 ml of which contains: 191 ml neurobasal medium, 4 ml B27 supplement (1 unit/ml; Gibco), 4 ml horse serum (PAA Laboratories), 500 µl 0.5 mm l-glutamine (Gibco), 100 µl 0.05% 2-mercaptoethanol (Gibco), 20 µl ciliarly neurotrophic factor (CNTF; 500 pg/ml; Alomone Labs), 2 µl glial-derived neurotrophic factor (GDNF; 100 pg/ml; Alomone Labs), 2 µl brain-derived neurotrophic factor (BDNF; 100 pg/ml), 2 ml P/S, in a 37°C, 5% CO_2_ humidified incubator for a minimum of 7 days.

### Mitochondrial function

Embryonic motor neurons (identified as having at least three processes) were loaded for 40 min with 20 nm TMRM (Molecular Probes) in a HEPES-buffered salt solution (HBSS) composed (mm): 156 NaCl, 3 KCl, 2MgSO_4_, 1.25 KH_2_PO_4_, 2 CaCl_2_, 10 glucose and 10 HEPES, pH adjusted to 7.3 with NaOH. The dye was present at the same concentration in all solutions throughout the experiment.

TMRM measurements were made using a Zeiss 710 CLSM equipped with a META detection system and a 40× oil immersion objective. Illumination intensity was kept to a minimum (at 0.1–0.2% of laser output) to avoid phototoxicity and the pinhole set to give an optical slice of ∼2 μm. TMRM was excited using the 565 nm laser line and fluorescence measured above 580. FAD^++^ autofluorescence was excited at 458 and measured at 510–530 nm.

The autofluorescence of NADH (and NAD(P)H) in motor neuron cultures was imaged using an epifluorescence inverted microscope equipped with a 40× fluorite objective. Excitation light was provided by a Xenon arc lamp, and the beam passed through a monochromator (Cairn Research, UK). Emitted fluorescence light was reflected through a long-pass filter to a cooled CCD camera (Retiga, QImaging, Canada) and digitized to 12 bit resolution. All imaging data were collected and analysed using software from Andor (UK). The blue autofluorescence emitted by the pyridine nucleotides NADH and NAD(P)H in their reduced form was excited with a 360 nm and emission was measured at 435–485 nm.

For assessment of the redox state, the dynamic range of NADH autofluorescence was measured. The dynamic range of the signals was defined by obtaining the maximally oxidized signal following the response to 1 μm trifluorocarbonylcyanide phenylhydrazone (FCCP, which stimulates maximal respiration and fully oxidizes the mitochondrial NADH pool). In these conditions, mitochondrial NADH is taken as 0%. The maximally reduced signal was then defined as the response to 1 mm sodium cyanide (NaCN) which fully inhibits respiration), preventing NADH oxidation, and so promoting maximal NADH reduction. In these conditions, mitochondrial NADH is taken as 100%.

All data presented were obtained from at least five cover-slips and two to three different cell preparations.

### Statistical analysis

An ANOVA test was used to compare between WT, *Zfp106^+/−^* and *Zfp106^−/−^* genotypes per time point followed by Bonferroni's multiple comparisons testing correction for weight, grip strength, rotarod, open field parameters distance moved and velocity, soleus muscle fibre types, and mitochondrial data. The Mann–Whitney test was used to compare WT with *Zfp106^−/−^* littermates per time point or *Zfp106^−/−^* cohorts between time points for TA and EDL maximum muscle force, surviving motor units innervating the EDL, FI and motor neuron survival. Cumulative frequencies for axon distribution were compared using the Kolmogorov–Smirnov test. Two-tailed tests were used in all instances and significance level was set at *P* < 0.05.

## Ethical approval

All experiments were performed under licence from the UK Home Office regulations. All applicable international, national and/or institutional guidelines for the care and use of animals were followed. All procedures performed in mice were in accordance with the ethical standards of the institutions or practice at which the studies were conducted.

## Supplementary Material

Supplementary material is available at *HMG* online.

## Funding

This work was funded by the UK Medical Research Council (MRC) to A.A.-A. and a Motor Neurone Disease Association (MNDA) project grant to A.A.-A. and EMCF. D.L.H.B. is a Wellcome Trust Senior Clinical Scientist Fellow and P.F. is a MRC/MNDA Lady Edith Wolfson Clinician Scientist Fellow. Funding to pay the Open Access publication charges for this article was provided by the MRC grant number: MC_UP_A390_1106.

## Supplementary Material

Supplementary Data
