# Supplementary material for: Deficiency of the zinc finger protein ZFP106 causes motor and sensory neurodegeneration
Source: Hum Mol Genet. 2015 Nov 24;25(2):291–307. doi: 10.1093/hmg/ddv471 (PMC4706115; doi:10.1093/hmg/ddv471)
Supplement: Supplementary Data [file supp_25_2_291__index.html]

Deficiency of the zinc finger protein ZFP106 causes motor and sensory neurodegeneration — Deficiency of the zinc finger protein ZFP106 causes motor and sensory neurodegeneration — Deficiency of the zinc finger protein ZFP106 causes motor and sensory neurodegeneration — Supplementary Data 

# Deficiency of the zinc finger protein ZFP106 causes motor and sensory neurodegeneration

## Supplementary Data

Supplementary Data

- Supplementary Data - Docx file
- Supplementary Figure 1 - tif file
- Supplementary Figure 2 - tif file
- Supplementary Figure 3 - tif file
- Supplementary Figure 4 - tif file
- Supplementary Figure 5 - tif file
- Supplementary movie1 - mp4 file
