# Supplementary material for: Deficiency of the zinc finger protein ZFP106 causes motor and sensory neurodegeneration
Source: Hum Mol Genet. 2015 Nov 24;25(2):291–307. doi: 10.1093/hmg/ddv471 (PMC4706115; doi:10.1093/hmg/ddv471)
Supplement: Supplementary Data [file supp_ddv471_ddv471supp.docx]

**Supporting Information**

**Figure S1.** *Zfp106* promoter expression patterns. (A) *LacZ* expression in 14.5 *Zfp106^+/-^* days post coitum mouse embryo, and in 7-week old *Zfp106^+/-^* (B) brain, (C) hind limb muscle, (D) muscle under the skin, and (E) the rib cage. *LacZ* expression is shown in blue. (F) qPCR analysis of a muscle enriched *Zfp106* transcript in TA muscle of 16-week old female WT and *Zfp106^-/-^* littermates (n = 5 per genotype); expression in *Zfp106^-/-^* mice is normalised to WT. p=0.05. (G) Northern blot analysis from brain and spinal cord RNA extracts revealed a dramatic reduction in the expression of *Zfp106* isoforms. Probe against *Zfp106* 3’UTR.

**Figure S2.**Behavioural analyses of *Zfp106^-/-^*males. (A) Male weights recorded weekly from ages 3 to 16 weeks; at least 5 mice were assessed per genotype per time point. Weight is diminished in *Zfp106^-/-^* male mice from 4 weeks of age (p < 0.05) and continues to significantly decrease, compared to WT and *Zfp106^+/-^*animals, to 16 weeks of age (p < 0.001). (B) Male grip strength and (C) accelerated Rotarod performance are reduced in *Zfp106^-/-^* mice at 6 and 13 weeks of age compared to WT and *Zfp106^+/-^* littermates (n ≥ 6 per genotype). (D,E) Open field assessment of (D) distance moved, and (E) velocity for male mice 14 weeks of age (See Materials and Methods). A reduction in distance moved (D), and velocity (E), was seen in 14-week old males *Zfp106^-/-^* mice when compared to WT littermates. p values are indicated (n ≥ 5 per genotype and time point). Numbers shown represent the mean ± SEM. *, p < 0.05; ** p < 0.001

**Figure S3**. Active cell death and decreased autophagosome numbers in *Zfp106^-/-^*spinal cords. (A,B) Representative images of TUNEL staining from lumbar L3-L4 region of *Zfp106*^-/-^(A) and WT littermate (B) at 16 weeks of age showing active cell death on going in the *Zfp106*^-/-^ section.

**Figure S4**. Axonal morphology at P9. A-F Representative pictures from semithin cross sections stained with toluidine blue from sciatic nerve (A, B), dorsal roots (C, D) and ventral roots (E, F) of WT and *Zfp106*^-/-^ mice. (G, H) Quantification of the total average g-ratio and axon diameter of WT and *Zfp106*^-/-^ mice (n= 3 animals/group) showing a significant decrease in axon diameter from the sciatic nerve. *p < 0.05. Results are reported as mean values +/- SEM.

**Figure S5.** Whole body and muscle abnormalities in *Zfp106^-/-^* mice. (A) Dorsal and ventral view of skinned *Zfp106^-/-^* and WT mice, at 14 weeks of age, showing much reduced muscle size. Length and width measurements given in cm. (B) Assessment of tibia length (mm) across three 14-week *Zfp106^-/-^* and WT mice; no significant difference seen. (C) Calculation of soleus fibre cross sectional area (log scale) from three 14-week old *Zfp106^-/-^* (1, 2 and 3) and three WT (1, 2 and 3) mice. Fibre type could not be determined for *Zfp106^-/-^* 1 and 2 mice. *Zfp106^-/-^* mice shower a greater variation in size of muscle fibres compared to WT soleus muscle. nd is not determined.

**Figure S6**. RNA-seq analysis. Spinal cords from 6-week-old *Zfp106^+/+^* and *Zfp106^-/-^* mice were analysed. (A) Heat map showing gene clustering from *Zfp106^+/+^* and *Zfp106^-/-^* spinal cords.

**Movie S1.** Progressive motor deficits of *Zfp106^-/-^* mice. A *Zfp106^-/-^* mouse at 6- and 15-weeks of age showing: (1) an age dependent increase in the number of errors made when walking across a wire grate; (2) an abnormal pulling in of all four limbs when held by tail; and (3) progressive gait abnormalities when placed in an arena.
