# Supplementary figures and images for: Deficiency of the zinc finger protein ZFP106 causes motor and sensory neurodegeneration
Source: Hum Mol Genet. 2015 Nov 24;25(2):291–307. doi: 10.1093/hmg/ddv471 (PMC4706115; doi:10.1093/hmg/ddv471)

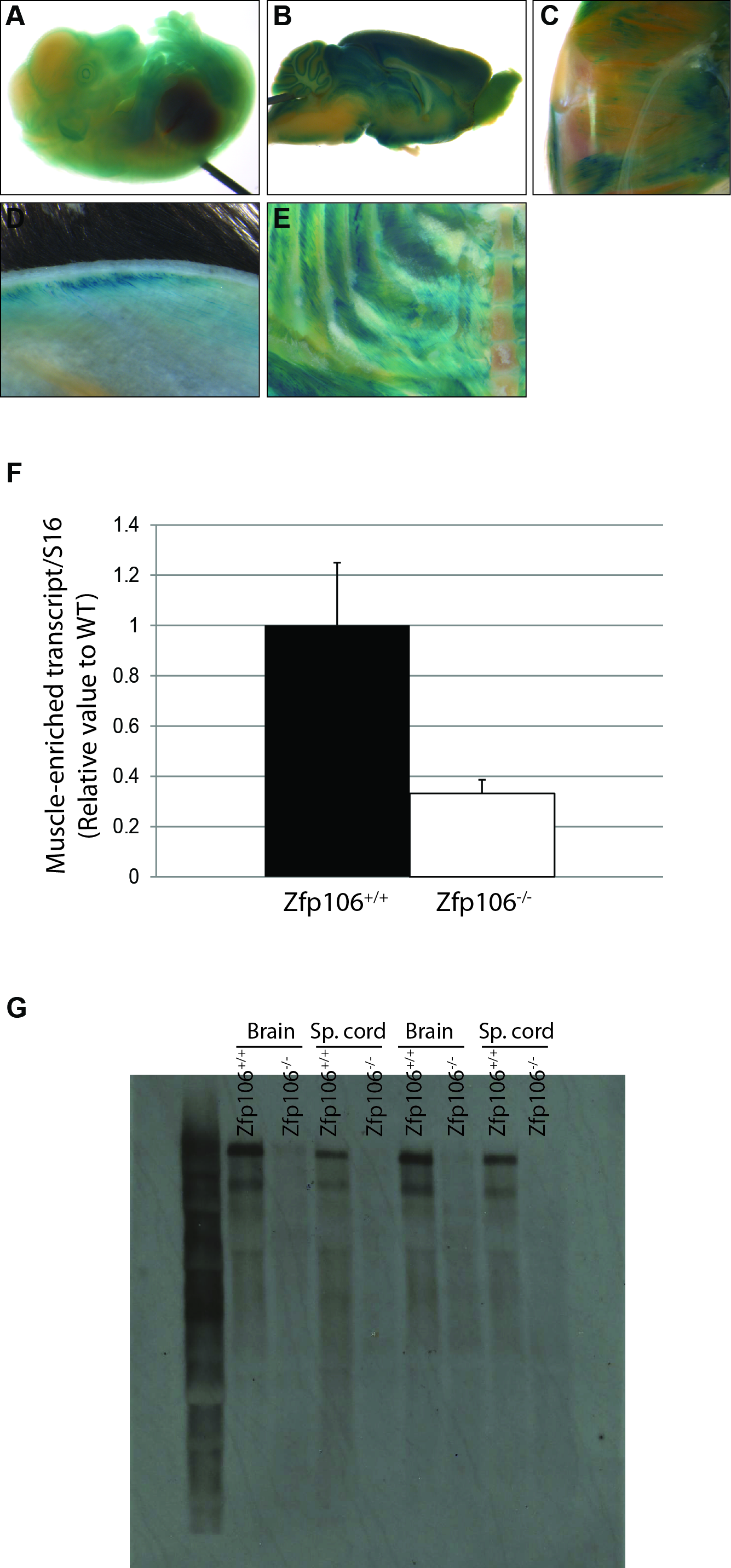

Supplement: Supplementary Data [file supp_ddv471_ddv471supp_fig1.tif]

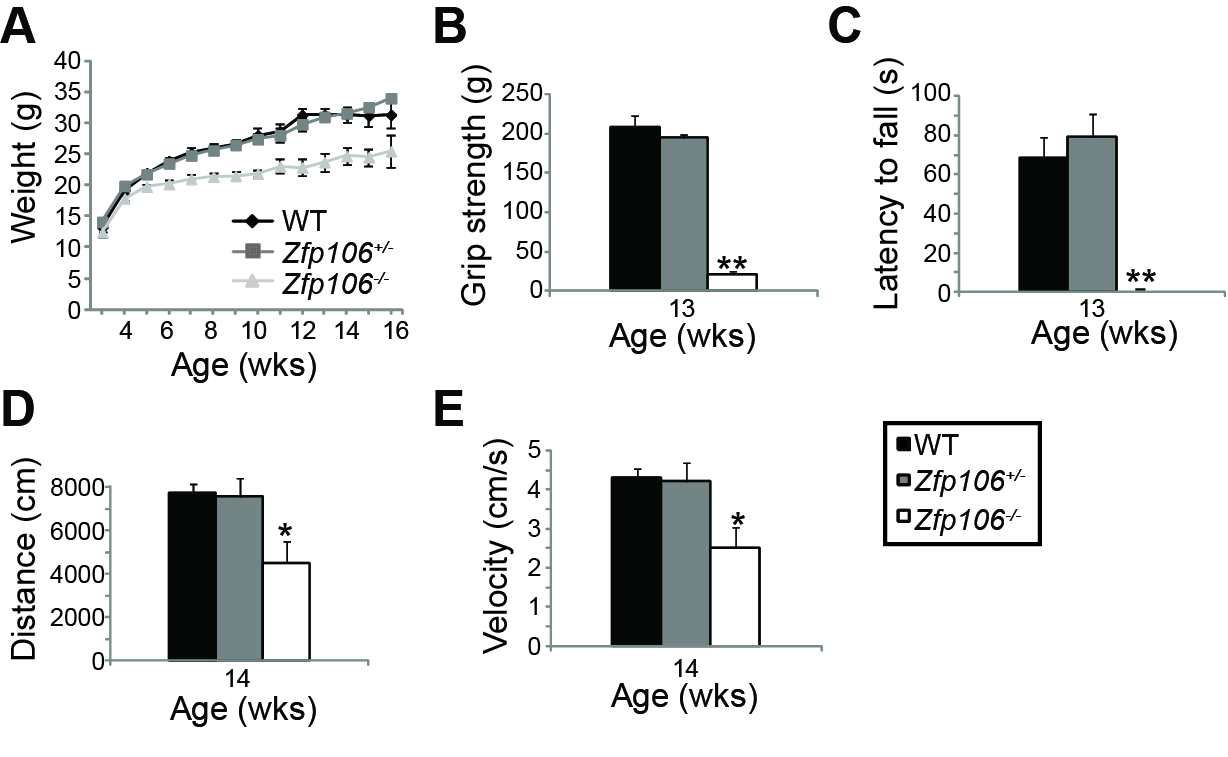

Supplement: Supplementary Data [file supp_ddv471_ddv471supp_fig2.tif]

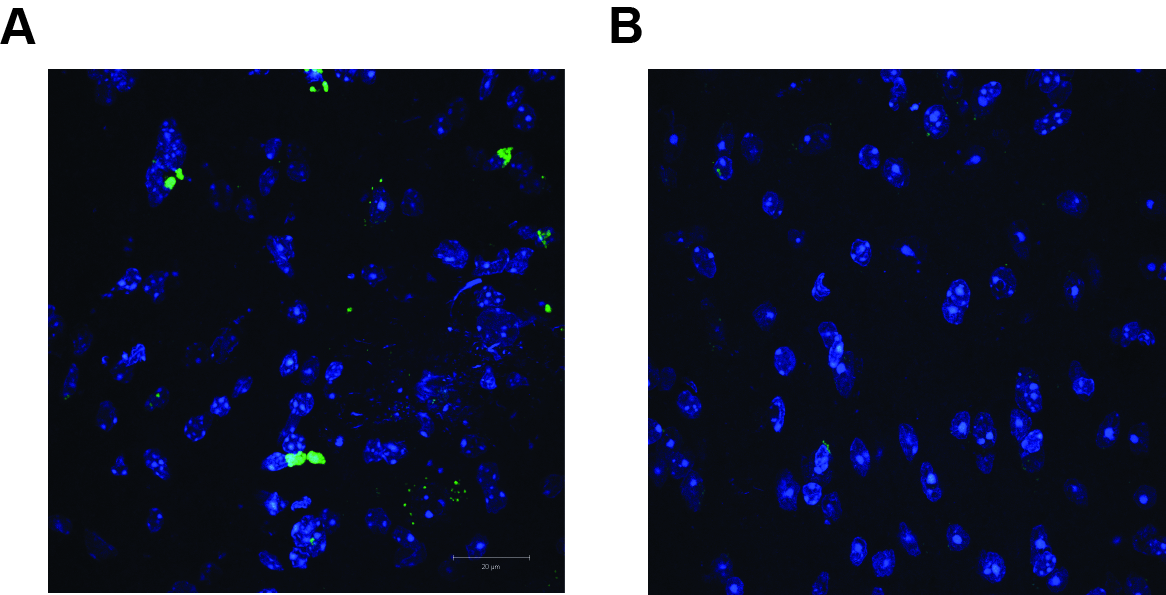

Supplement: Supplementary Data [file supp_ddv471_ddv471supp_fig3.tif]

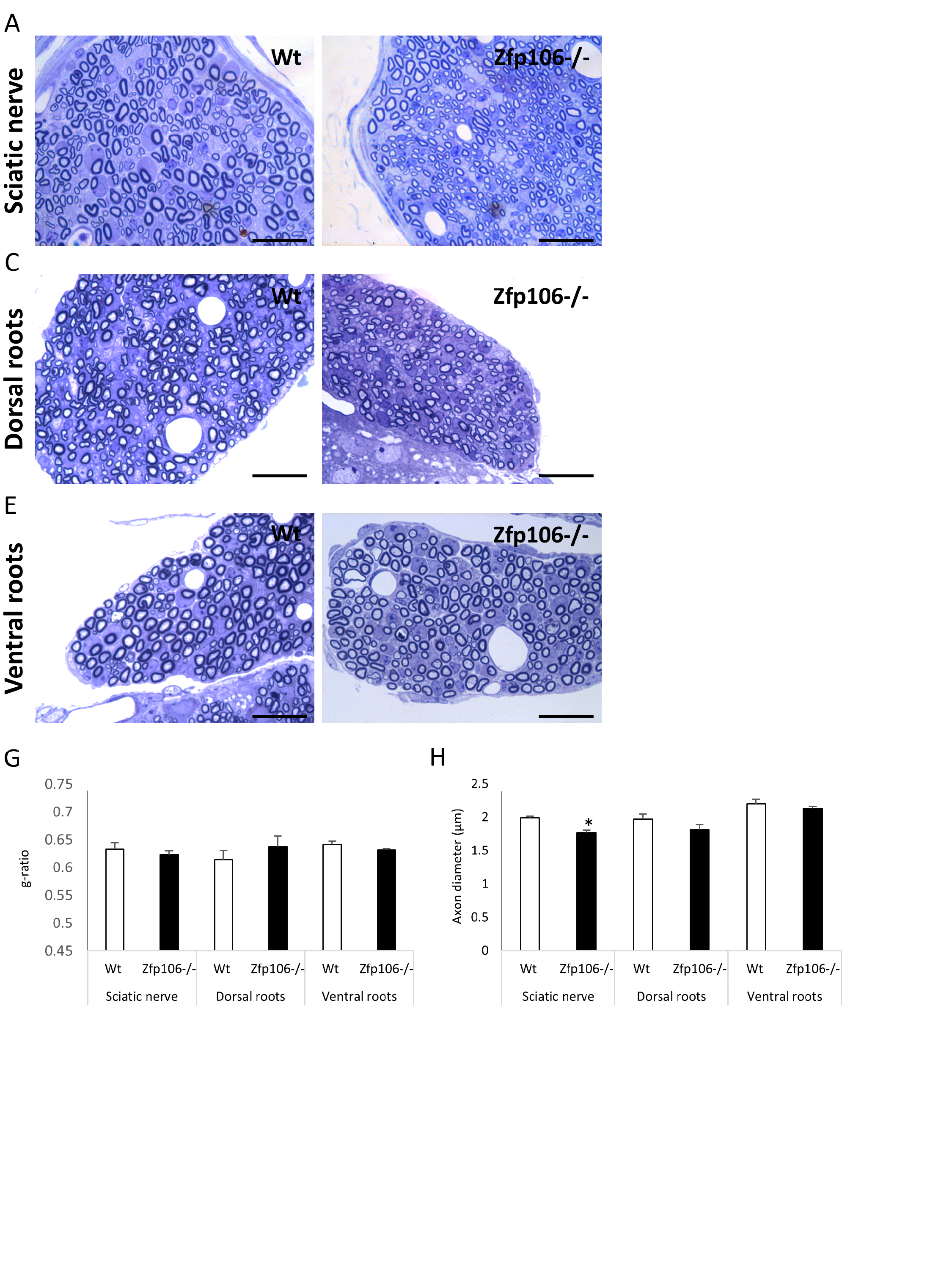

Supplement: Supplementary Data [file supp_ddv471_ddv471supp_fig4.tif]

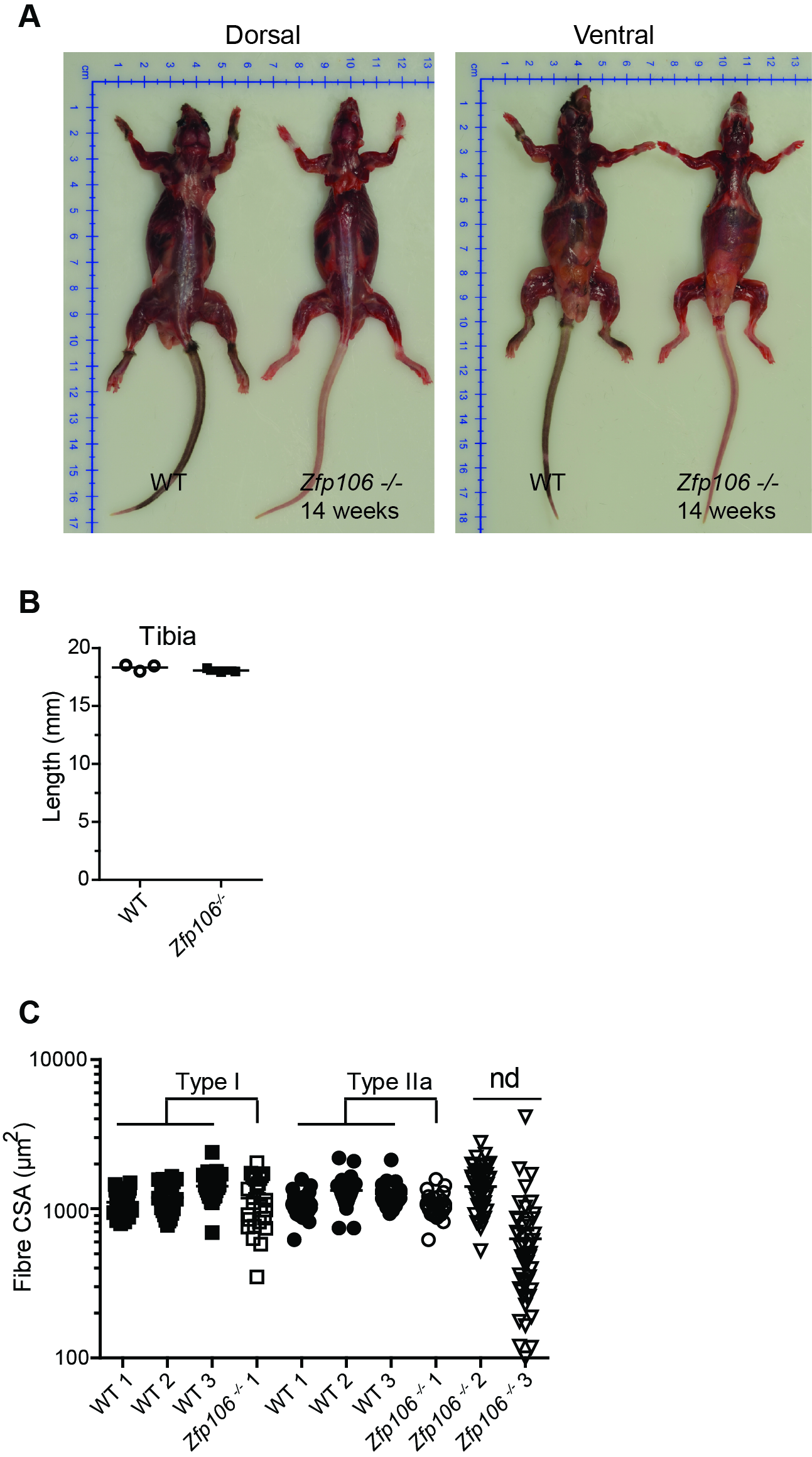

Supplement: Supplementary Data [file supp_ddv471_ddv471supp_fig5.tif]
